# Assessment of single-vessel cerebral blood velocity by phase contrast fMRI

**DOI:** 10.1371/journal.pbio.3000923

**Published:** 2021-09-09

**Authors:** Xuming Chen, Yuanyuan Jiang, Sangcheon Choi, Rolf Pohmann, Klaus Scheffler, David Kleinfeld, Xin Yu

**Affiliations:** 1 High-Field Magnetic Resonance, Max Planck Institute for Biological Cybernetics, Tübingen, Germany; 2 Department of Neurology, Wuhan University, Renmin Hospital, Wuhan, China; 3 Athinoula A. Martinos Center for Biomedical Imaging, Massachusetts General Hospital and Harvard Medical School, Charlestown, Massachusetts, United States of America; 4 Graduate Training Centre of Neuroscience, International Max Planck Research School, University of Tübingen, Tübingen, Germany; 5 Department for Biomedical Magnetic Resonance, University of Tübingen, Tübingen, Germany; 6 Department of Physics, University of California at San Diego, La Jolla, California, United States of America; 7 Section of Neurobiology, University of California at San Diego, La Jolla, California, United States of America; Columbia University, UNITED STATES

## Abstract

Current approaches to high-field functional MRI (fMRI) provide 2 means to map hemodynamics at the level of single vessels in the brain. One is through changes in deoxyhemoglobin in venules, i.e., blood oxygenation level–dependent (BOLD) fMRI, while the second is through changes in arteriole diameter, i.e., cerebral blood volume (CBV) fMRI. Here, we introduce cerebral blood flow–related velocity-based fMRI, denoted CBFv-fMRI, which uses high-resolution phase contrast (PC) MRI to form velocity measurements of flow. We use CBFv-fMRI in measure changes in blood velocity in single penetrating microvessels across rat parietal cortex. In contrast to the venule-dominated BOLD and arteriole-dominated CBV fMRI signals, CBFv-fMRI is comparable from both arterioles and venules. A single fMRI platform is used to map changes in blood pO_2_ (BOLD), volume (CBV), and velocity (CBFv). This combined high-resolution single-vessel fMRI mapping scheme enables vessel-specific hemodynamic mapping in animal models of normal and diseased states and further has translational potential to map vascular dementia in diseased or injured human brains with ultra–high-field fMRI.

## Abstract 2

This study presents a phase contrast-based, high field MRI-based approach for the functional mapping of cerebral blood velocity in individual cortical arterioles and venules in the rat cortex; this approach can be combined with previously established approaches to map BOLD, CBV, and blood velocity from penetrating microvessels.

## Introduction

Cerebral blood flow (CBF) is a key hemodynamic readout coupled to neuronal dynamics and viability in normal and diseased brain states [1]. Changes in CBF may be monitored directly within individual blood vessels through the use of optical-based particle tracking techniques [2]. A variety of imaging methods have been developed to measure CBF across multiple spatial scales, from capillary beds up through brain-wide vascular networks in animal brains. These include in vivo multiphoton microscopy [3], optical coherence tomography [4], optoacoustic imaging [5], and laser doppler and speckle imaging [6,7]. In particular, doppler based functional ultrasound imaging provides a unique advantage to detect the CBF in the brain with a high spatiotemporal resolution, which can be readily applied for awake animal imaging [8–10]. However, the spectrum-specific signal transmission in ultrasound methods cannot effectively pass the skull of animals without significant degradation of the signal-to-noise ratio (SNR). Typically, a craniotomy or a procedure to thin the skull is needed to detect the hemodynamic signal [2]. While current techniques support transcranial imaging into the superficial layers of the cortex [11–13], only functional MRI (fMRI) provides a noninvasive approach for measuring hemodynamic signals throughout the brain.

Changes in CBF may be detected by fMRI that is based on arterial spin labeling (ASL), in which water protons in a major upstream vessel are spin-polarized with an additional radio frequency (RF) B1 field [14–16]. The ASL-based CBF fMRI technique detects local changes in the flow of blood through brain tissue [17]. Two other fMRI-based techniques provide indirect information about changes in CBF. Blood oxygenation level–dependent (BOLD) fMRI is used to determine changes in the ratio of deoxy to oxyhemoglobin in the blood and is an indirect measure of changes in brain metabolism [14,18,19]. Cerebral blood volume (CBV) fMRI is used to measure changes in blood volume, i.e., essentially changes the diameter of arterioles, based on the use of exogenous contrast agents, e.g. monocrystalline iron oxide nanoparticle (MION), or the endogenous vascular-space-occupancy (VASO) mapping scheme to differentiate blood from brain tissue [20–23].

In contrast to the ASL-based orientation-specific flow measurement [24–27], phase contrast (PC) MRI relies on gradient-oriented dephasing of magnetized protons to map the velocity, i.e., direction and speed, of blood flow [28,29]. Past work with 7 T MRI showed that PC-MRI can be used to measure flow in the perforating arteries through the white matter and the lenticulostriate arteries in the basal ganglia of human brains [30–33]. However, the SNR was insufficient in these prior studies to map changes in flow, and thus changes in CBF, concurrent with changes in neuronal activation.

Here, we report on a PC-MRI method to detect vessel-specific changes in blood velocity on the timescale of single trials. We denote this method cerebral blood flow–related velocity, abbreviated CBFv. We build on past implementations of PC-MRI [30,34–37]. Here, we utilize a small surface RF coil with high-field MRI, i.e., 14.1 T, for improved SNR. This advance allows us to map the BOLD, CBV, and CBFv-fMRI signals from individual penetrating venules and arterioles, which span 20 to 70 μm diameter, with high spatial resolution [22,38,39].

## Results

### Phantom validation of high-resolution PC-based flow velocity measurement

**Fig 1:**
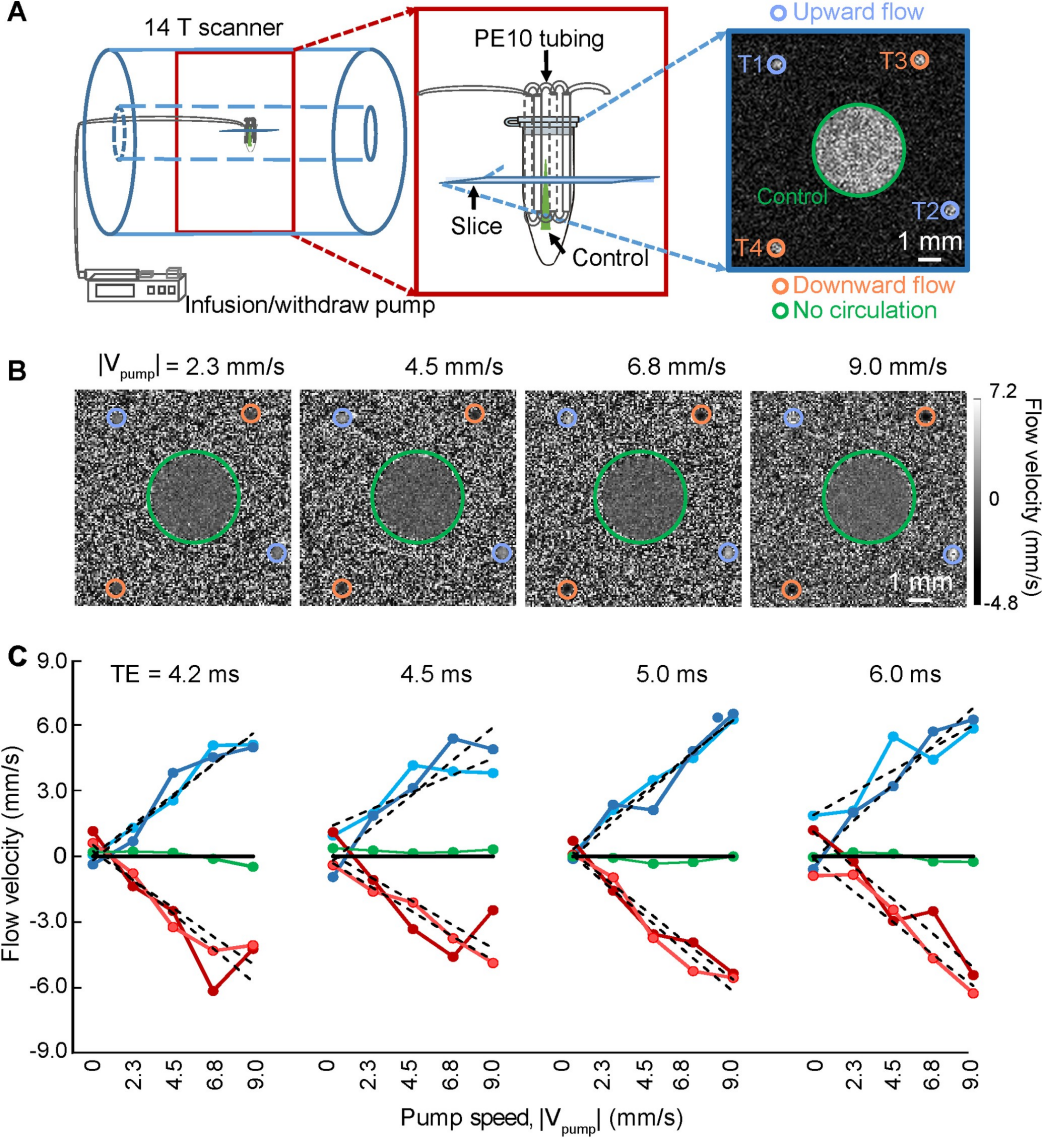
In vitro flow velocity measurements with PC-MRI. **(A)** Schematic drawing of the phantom experimental flow chamber in the 14.1 T scanner. An expanded image (red box) shows the circulatory system constructed of capillary tubes. A representative FLASH MRI image (blue box), 500 μm in thickness, shows the capillary positions. ROIs T1 and T2, contoured in purple, indicate the upward flow. ROIs T3 and T4, in orange contour, indicate the downward flow. The green contour indicates the stagnant fluid. **(B)** Representative images with different flow velocity in the capillaries T1 to T4 in panel A. TE = 5.0 ms for all panels. **(C)** The plot of flow velocity estimates from the 5 ROIs with different TEs, as marked, and different pump rates, as indicated and marked in panel B. The dotted lines correspond to a linear fitting for velocity measurements of different ROIs. The data underlying this figure can be found in S1 Data. FLASH, fast low angle shot; PC, phase contrast; ROI, region of interest; TE, echo time.

**Fig 2:**
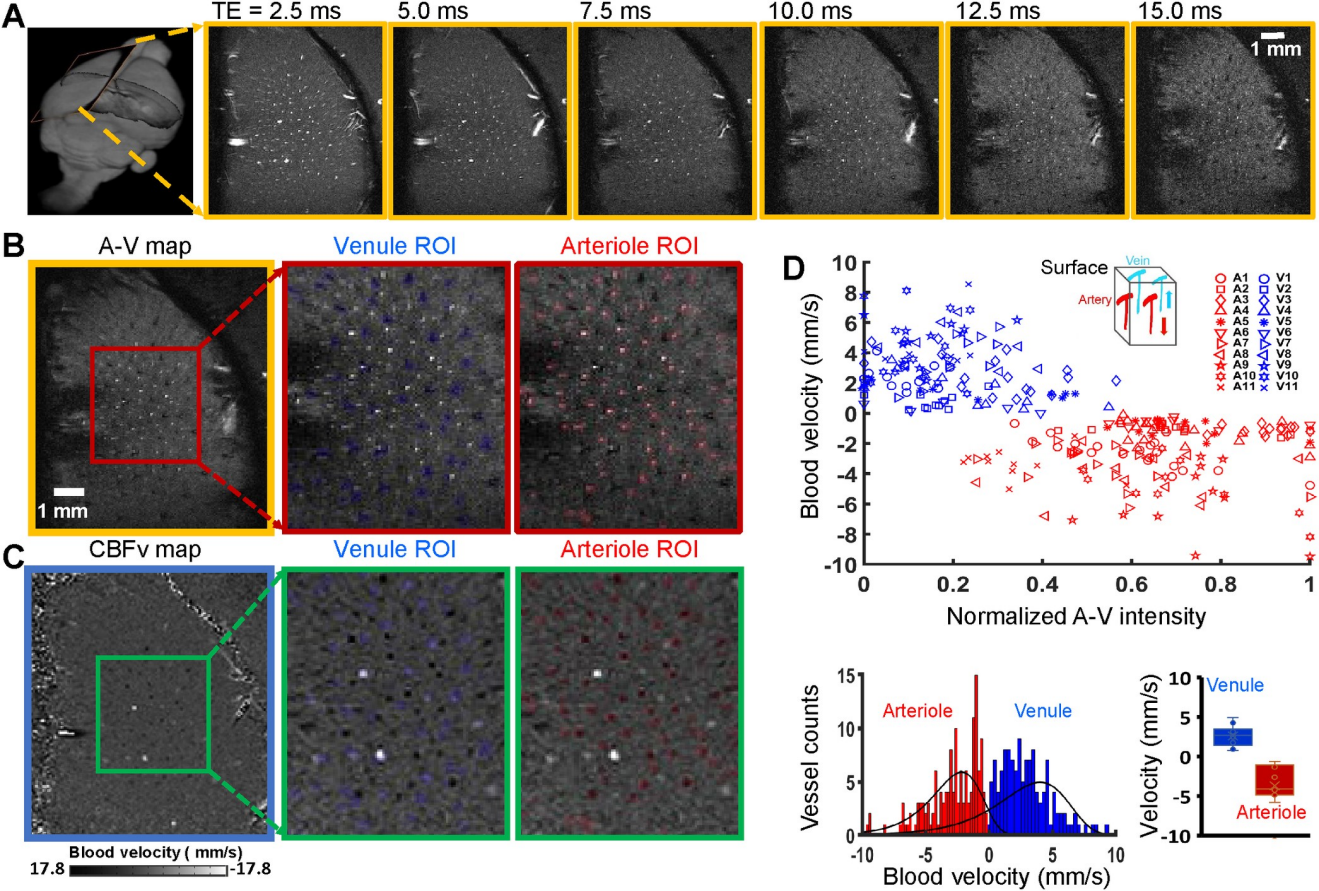
PC-based single-vessel flow velocity (CBFv) mapping. **(A)** Representative 2D MGE slices (yellow boxes) from a deep layer of the primary forepaw somatosensory cortex (first frame) at different TEs, as indicated. **(B)** The 2D A–V map (yellow box) derived from the images with different TEs in panel A, arterioles, and venules appear as bright and dark voxels, respectively. The expanded views (red boxes) show individual venules, i.e., black voxels marked in blue, and arterioles, i.e., white voxels marked in red. **(C)** The vectorized flow velocity map (blue box) from the same 2D MGE slice in panel B. The expanded views (green boxes) show the individual venules, i.e., white dots with positive velocity, and arterioles, i.e., black dots with negative velocity. Note that 2 bright dots are caused by the “overflowed” velocity beyond the maximal velocity, i.e., the Venc parameter, defined in the PC-MRI sequence, which could be not correctly estimated. **(D)** Scatter plot of the flow velocities from individual arterioles and venules as the function of the normalized signal intensities of each vessel in the A–V map of panel B, data from 11 rats as indicated. Insert shows the blood flow direction of arterioles and venules in the forepaw somatosensory cortical region. The lower panel shows the histogram of the blood velocity distribution across arterioles and venules, as well as the bar graph to show the mean velocity. The data underlying this figure can be found in S2 Data. A–V, arteriole–venule; CBFv, cerebral blood flow–related velocity; MGE, multi-gradient echo; PC, phase contrast; ROI, region of interest; Venc, velocity encoding; TE, echo time.

For calibration, we constructed an in vitro capillary tubing circulatory system to mimic penetrating vessels, with flow rates from 1 to 10 mm/s (**Fig 1A**). A 2D PC-MRI slice is aligned perpendicular to the capillary tubing (**Fig 1A** and **1B)** and provides a voxel-specific measurement of the flow velocity through 2 tubes with the upward flow (positive sign, bright dots in **Fig 1B**) and 2 tubes with the downward flow (negative sign, dark dots in **Fig 1B**), as well as a control tube. We observe a monotonic and near linear relation between the velocity measured by PC-MRI and the true velocity: V_meas_ = (0.67 ± 0.01) _Vpump_ + (0.02 ± 0.11) mm/s at echo time (TE) = 5.0 ms (**Fig 1C**). The small offset could be caused by eddy current effects and other gradient-related scaling errors of the PC-MRI sequence [40–42]. We further observe that the measured velocities are relatively insensitive to the value of TE (**Fig 1C**).

We implemented the high-resolution PC-MRI with 14.1 T MR scanning for in vivo measurement of blood flow from individual penetrating arterioles and venules. We collected data throughout the infragranular cortex, i.e., layer V, of anesthetized rats. A surface RF transceiver coil with 6-mm diameter was constructed and attached to the rat skull (**S1 Fig**) to improve the SNR of PC-MRI images as well as multi-gradient echo (MGE) images used for arteriole–venule (A–V) mapping [22,38]. This coil was essential for the high-resolution mapping with a fast sampling rate of the single-vessel flow velocity over a complete hemisphere of the rat brain (**Figs 2A and S1E**).

### In vivo PC-based flow velocity mapping of penetrating microvessels

We first acquired the single-vessel A–V map by aligning a 500-μm thick 2D MRI slice perpendicular to penetrating vessels through layer V of one hemisphere (**Fig 2A** and **2B**). We designed the pulse sequence for PC-MRI to achieve the same slice geometry of the A–V map so that the CBF deduced from PC-MRI signals could be overlaid with individual penetrating arterioles and venules in the single-vessel flow velocity map (**Fig 2A** and **2C**). The arteriole blood flows into the cortex, while the venule blood flows outward, which determines the sign of the flow velocity. Vessel-specific velocities were plotted as a function of the normalized signal intensity in the A–V map and corroborated our ability to determine flow velocity specific to arterioles and venules (**Fig 2C** and **2D**). The measured flow velocities range from 1 to 10 mm/s, as previously measured with optical methods [43,44]. To probe the reliability of the single-vessel MR-based flow velocity method, we compared the velocities detected by PC-MRI methods with different TEs and flip angles and observed comparable results across a range of parameters (**S2 Fig**). It should be noted that altered vessel velocities detected from the same animal depend on the vessel sizes and orientation angles relative to the 2D slice. Lastly, the large variability of blood velocity across different animals could be caused by the varied physiological states of animals under anesthesia, as well as by degraded gradient performance during high duty cycle PC-MRI; see optimization step in Methods. All told, these data demonstrate the feasibility of in vivo PC-based blood velocity mapping from individual penetrating arteriole and venules.

### PC-based CBFv-fMRI from individual arterioles and venules

**Fig 3:**
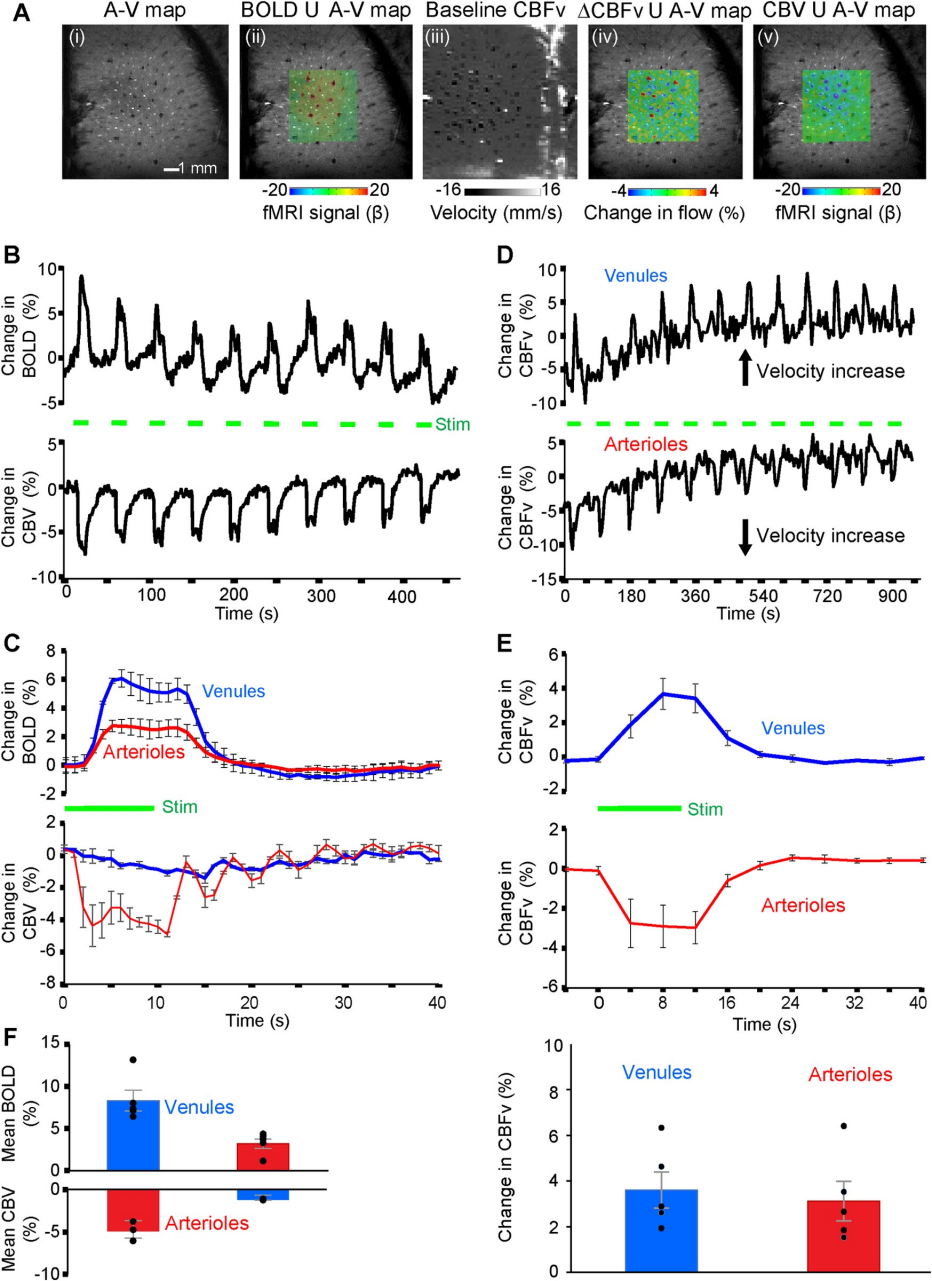
Maps of task-related hemodynamic signals with single-vessel BOLD, CBV, and CBFv-fMRI. **(A)** Different MRI measurement strategies on the same 2D slice. From left to right: (i) the A–V map defines arterioles as bright dots and venules as dark dots; (ii) the evoked bSSFP-based BOLD fMRI map, within a green subregion, on top of the A–V map; (iii) the PC-MRI map of baseline CBFv; (iv) the CBFv-fMRI map on top of the A–V map with an increased flow velocity corresponding to brighter voxels for venules and darker voxels for arterioles; and (v) evoked bSSFP-based CBV-fMRI map on top of the A–V map. **(B)** The time courses of evoked bSSFP-BOLD and CBV-fMRI with the block design paradigm from venules and arterioles shown in panel A. Forepaw stimulation pulse of 330 μs in width and 1 mA in amplitude delivered at 3 Hz for 10 seconds. **(C)** Averaged time courses of the fractional change for evoked BOLD and CBV signals from venule and arteriole ROIs of different rats (mean ± SEM, the green bar shows stimulation duration). **(D)** The time courses of the evoked CBFv changes from the arteriole and venule ROIs show increased velocity from both arterioles and venules with the block design, 10-second duration stimulation paradigm. **(E)** The averaged time courses of the evoked CBFv changes show the velocity increase from both arteriole and venule ROIs with the block design stimulation paradigm from 5 rats (mean ± SEM, the green bar shows stimulation duration). **(F)** Bar graph shows that peak BOLD changes of venule are significantly higher than those of arteriole (5 rats, *p* = 0.009), while the peak CBV changes of arteriole are significantly higher than those of venule (3 rats, *p* = 0.028). In contrast, the peak CBFv changes are only slightly, but not significantly, higher in venules than arterioles (5 rats, *p* = 0.063). The data underlying this figure can be found in S3 Data. A–V, arteriole–venule; BOLD, blood oxygenation level–dependent; bSSFP, balanced steady-state free precession; CBFv, cerebral blood flow–related velocity; CBV, cerebral blood volume; ROI, region of interest.

We contrasted the complementary capabilities of PC-based CBFv-fMRI against the signals observed with the balanced steady-state free precession (bSSFP)-based single-vessel BOLD- and CBV-fMRI mapping method [38] (**Fig 3**). We first created an A–V map through the infragranular layers of the forepaw region of the primary somatosensory cortex (**Fig 3Ai**), followed by 2D-bSSFP to detect stimulus-induced changes in the single-vessel BOLD fMRI signal (**Fig 3Aii**). We next performed single-vessel PC-MRI flow velocity measurements to measure baseline flow in penetrating arterioles and venules, using 100 × 100 μm^2^ in-plane resolution, a sampling rate of 4-second repetition time (TR) per image and the same geometry as the 2D-bSSFP method (**Fig 3Aiii**). Changes in CBFv upon stimulation overlapped with individual penetrating vessels in the A–V map (**Fig 3Aiv**). Lastly, we performed 2D-bSSFP for single-vessel CBV-fMRI mapping by intravenous injection of iron particles into the blood in the same animals (**Fig 3Av**). The BOLD fMRI signal is primarily detected from individual penetrating venules, while the CBV-weighted signal is mainly located at the individual penetrating arterioles (dark dots in **Fig 3Aii** with bright dots in **Fig 3Av**). In contrast, the CBFv-fMRI signal is observed in both the penetrating arterioles and venules (**Fig 3Aiv**).

The stimulus-evoked responses of all CBV, BOLD, and CBFv-fMRI signals were studied with an on/off block design (**Fig 3B–3E**). Group analysis shows that the positive BOLD signal from venule voxels is significantly higher than the arteriole-specific BOLD signal (**Fig 3C**). In contrast, the arteriole dilation leads to an earlier CBV-weighted negative fMRI signal, which is much stronger and faster than the signal from passive venule dilation (**Fig 3C**), as expected [45–47]. Group analysis shows the similar temporal dynamics of CBFv changes, but with different signs in arterioles and venules (**Fig 3D**). In contrast to the venule-dominated BOLD and arteriole-dominated CBV responses (**Fig 3C** and **3F**), the percentage changes in CBFv-fMRI is statistically indistinguishable between arterioles (3.12 ± 0.87%) and venules (3.60 ± 0.80%) (**Fig 3E** and **3F**). The voxel-wise hemodynamic changes of BOLD, CBV, and CBFv are illustrated in **S3 Fig** and **S1 Movie**.

## Discussion

Despite the plethera of existing tools developed for CBF measurements in both animal and human brains, it remains challenging to noninvasively detect the flow dynamics of intracortical microvessels. Here, we optimized PC-MRI to map the vectorized single-vessel flow velocity of penetrating arterioles and venules and further developed the single-vessel CBFv-fMRI technique to directly measure flow velocity in rat brains. In combination with previously established single-vessel BOLD- and CBV-fMRI methods, the PC-based single-vessel CBFv-fMRI method provides complementary information to map vessel-specific hemodynamic responses with high-resolution fMRI.

In contrast to the conventional ASL methods, PC-based MRI mapping allows arterioles and venules to be distinguished for simultaneous velocity measurements through a 2D plane. Also, ASL has less vascular specificity because water exchange through the blood–brain barrier of capillary beds increases the weighting of the ASL-based flow signal for parenchyma voxels [48,49]. Furthermore, there is significant variability in the transit time to flow from arterioles to venules through the capillary bed [50], which complicates the distinction of arterioles and venules by simple ASL-based CBF mapping. We detected the velocity from penetrating microvessels in the deep cortical layers with PC-MRI, with velocity values from 1 to 10 mm/s (**Fig 2**). It is noteworthy that the PC-based vessel velocity measurement is based on measuring water protons in blood but not limited to the flow of red blood cells. Still, the PC-based velocity from microvessels matches well with the previous optical measurement [2,43,44]. We conclude that high-resolution PC-MRI is ideal for noninvasive single-vessel CBFv-fMRI mapping.

The noninvasive measurement of microvessel blood velocity changes coupled to neuronal activation is a critical step to elucidate the neurovascular coupling mechanism underlying a variety of brain disorders in animal models. Existing methods, e.g., multiphoton microscopy [3,51,52] or doppler based ultrasound imaging [8–10], enable the detection of red blood cell velocity or CBF/CBV from microvessels with ultra-high resolution. Yet ultrasound requires a craniotomy or thinned skull [53] for effective spectrum-specific signal transmission through deep cortical layers or subcortical regions in rats and other animals. Such a surgical procedure has been well documented to induce perturbation of the physiological microenvironment in the brain [54–56], which potentially confounds the disease-related functional CBF measurements in animal brains. In contrast, conventional MRI methods, including PC-MRI, mainly detect blood flow or velocity in large vessels and seldom identify the functional CBFv of penetrating microvessels in the brain. By implementing a local RF coil with the high-field MR scanner, our work showed the feasibility to map the velocity changes from individual penetrating arterioles and venules with diameter 20 to 70 μm [22,38].

The novel applications of PC-based CBFv-fMRI can be divided into 2 different directions. First, the PC-based CBFv-fMRI provides a possible scheme to be combined with single-vessel BOLD and CBV fMRI signals for quantitative assessment of the cerebral metabolic rate of oxygen utilization in relation to flow [57]. These vessel-specific hemodynamic measurements can then be used to potentially validate the calibrated BOLD model [58] in future studies. Secondly, we now have a means to test the resting-state single-vessel correlation patterns of CBFv signals from penetrating arterioles and venules (**S4 Fig**). In contrast to the T2*-weighted resting-state readout of BOLD or CBV signal fluctuation [38], which amplifies the contrast-to-noise ratio (CNR) from the vessel-specific oxygenation or vessel diameter changes, CBFv signal fluctuation from penetrating microvessels provides a direct measurement of blood velocity changes across a large field of view. The measurement of PC-based CBFv signal fluctuation from microvessels can be further combined with single-vessel CBV-fMRI to study the vascular dynamic mechanism that underlies vasomotion-mediated perivascular clearance in animal models with Alzheimer disease (AD) [59,60]. In particular, both vasomotion-based ateriole diameter changes, i.e., the CBV signal fluctuation, and corresponding blood velocity changes, i.e., CBFv signal fluctuation, can be mapped across hippocampal penetrating vessels. This may be further combined with simultaneous fiber photometry based fluorescent recordings from genetically encoded biosensors [39] to identify the pathological vasodynamics in transgenic AD animals.

The accurate measurement of PC-based CBFv from microvessels relies on multiple factors. First, a remaining complication with PC-MRI mapping is the presence of small offsets in velocity as shown in our phantom capillary tubing studies with circulating flow under different conditions. The phase-dependent velocity encoding depends on the quality of the magnetic field gradients, and mismatched eddy currents of multiple gradients with opposite polarities, as well as the nonlinear and distorted gradient fields, could contribute to distortions in gradients [40–42]. We have described a list of parameters that should be optimized when implementing high-resolution PC-based CBFv measurements (Methods). Also, the high-resolution PC-MRI method is a high duty cycle sequence and slight heating of the gradient coil during scanning may alter the gradient performance, consistent with the baseline drift of the CBFv-fMRI signal in the first 5 minutes of scanning (**Fig 3D**). Nevertheless, it should be noted that the percentage velocity changes from individual arterioles and venules can be readily detected with the PC-based CBFv-fMRI measurement regardless of the gradient heating–related baseline drift. Another factor that contributes to the phase-dependent velocity error originates from the limited spatial resolution of PC-MRI images in comparison to the diameters of small vessels, i.e., the partial volume effect [36], although corrections are possible [30,37].

The present work shows the feasibility of PC-based CBFv-fMRI in rat brains using a ultra–high-field strength MR scanner (14 T). It should be noted that the high-resolution PC-based CBFv measurement can also be applied with a 9.4 T scanner to detect the microvessel blood velocity at a similar scale to 14 T measurement (**S5 Fig**). This supports a broader usage of noninvasive animal CBFv mapping. The translational potential of the PC-based CBFv-fMRI remains to be investigated. Previously, we detected low-frequency fluctuation of single-vessel resting state BOLD fMRI signals (TR = 1 second) from individual sulcus veins and arteries in the occipital lobe of the human brain [38]. The T2*-weighted BOLD resting state fMRI signal fluctuation from individual vessels can be separated from noise artifacts due to pulsation or other motion effects. In contrast, pulsation could be a significant confounding issue for PC-based blood velocity mapping from individual vessels [61,62]. To better differentiate the pulsational contribution to CBFv signal fluctuation given different frequency ranges, we will need to increase the sampling rate by implementing phased-array surface coils for focal field of view measurement and an accelerated PC-MRI sequence.

## Methods

### Design of a phantom capillary tubing flow system

To validate the PC-MRI sequence, a plastic circulatory flow phantom composed of the capillary tubing (PE-10, Instech Laboratories, (Plymouth Meeting, PA, USA) inner diameter 210 μm) was constructed to mimic the geometry of cortical blood vessels (**Fig 1A**). The capillary tube was connected to a programmable syringe infusion/withdraw pump (Pump Elite 11, Harvard Apparatus, Holliston, MA, USA) with an infusion rate of 0.25, 0.5, 1.0, 1.5, and 2.0 mL/h, which were transferred to the flow velocity of the capillary tubing as shown in **Fig 1**. We infused manganese solution (50 mM MnCl_2_, Sigma-Aldrich, Germany) through the tubing. The phantom capillary tubing was immersed in Fomblin (Sigma-Aldrich, Germany) to avoid air interface artifacts.

### Animal preparation for fMRI

All surgical and experimental procedures were approved by local authorities (Regierungspraesidium, Tübingen Referat 35, Veterinärwesen, Leiter Dr. Maas, protocol #KY 2/14 2/17) and were in full compliance with the guidelines of the European Community (EUVD 86/609/EEC) and guidelines of the Animal Care and Use Committee and the Animal Health and Care Section of Massachusetts General Hospital. The experimental animals were Sprague-Dawley male rats, approximately 250 g, provided by the Charles River Laboratories. A total of 25 rats were used in all experiments (the evoked bSSFP-BOLD/CBV and PC-MRI signals were acquired from 5 of 25 rats).

Detailed surgical procedures have been described previously [38,39]. Briefly, rats were first anesthetized with isoflurane, 5% (v/v) induction, and 1% to 2% (v/v) maintenance, and each rat was orally intubated with a mechanical ventilator (SAR-830, CWE, PA, USA). The femoral artery and vein were catheterized with plastic catheters (PE-50, Instech Laboratories) to monitor the arterial blood gas, administrate drugs, and constantly measure the blood pressure. After catheterization, rats were secured in a stereotaxic apparatus, and a custom-made RF coil was fixed above the skull with cyanoacrylate glue (454, Loctite, Henkel, Dusseldorf, Germany). After surgery, isoflurane was switched off and a bolus of α-chloralose (80 mg/kg, Sigma-Aldrich, Germany) was intravenously injected. A mixture of α-chloralose (26.5 mg/kg/h) and the muscle relaxant (pancuronium bromide, 2 mg/kg/h) was continuously infused to maintain the anesthesia and minimize motion artifacts. Throughout the whole experiment, the rectal temperature of rats was maintained at 37°C by using a feedback heating system. All relevant physiological parameters were constantly monitored during imaging, including heart rate, rectal temperature, arterial blood pressure, the pressure of the tidal ventilation, and end-tidal CO_2_. Arterial blood gases were checked to guide the physiological status adjustments by changing the respiratory volume or administering sodium bicarbonate (NaBic 8.4%, Braun, Melsungen, Germany) to maintain normal pH levels. Dextran-coated iron oxide particles (15 to 20 mg of Fe/kg, BioPAL, Massachusetts, USA) were intravenously injected for CBV-weighted signal acquisition.

### fMRI setup

All images were acquired with a 14.1 T, 26 cm horizontal bore magnet (Magnex Scientific, Oxford, UK) interfaced through the Bruker Advance III console (Bruker, Billerica, Massachusetts, USA). The scanner is equipped with a 12-cm magnet gradient set capable of providing a strength of 100 G/cm and a 150 μs rise time (Resonance Research, Billerica, MA, USA). We also applied the 9.4 T scanner (Bruker) with a 21-cm bore size (Magnex Scientific) to test the PC-MRI sequence. A custom-made transceiver coil with an internal diameter of 6 mm was used for fMRI image acquisition. For the electrical stimulation, 2 custom-made needle electrodes were placed on the forepaw area of the rats to deliver the electrical pulse sequences; 330 μs duration at 1.0 ~ 2.0 mA repeated at 3 Hz for 10 seconds using a stimulus isolator (A365, WPI, Sarasota, FL, USA). The stimulation duration and frequency were triggered directly through the MRI scanner which was controlled by Master-9 A.M.P.I. system (Jerusalem, Israel). The triggering pulses from the MRI scanner were also recorded by the Biopac system (MP150, Biopac Systems, USA).

### Single-vessel MGE imaging

To anatomically map individual arterioles and venules penetrating deep cortical layers of the somatosensory cortex, a 2D MGE sequence was applied with the following parameters: TR = 50 ms; TE = 2.5, 5.0, 7.5, 10.0, 12.5, and 15.0 ms; flip angle = 55°; matrix = 192 × 192; in-plane resolution = 50 × 50 μm^2^; slice thickness = 500 μm. The A–V map was made by averaging MGE images from the second echo to the fifth echo. In the A–V map, the arteriole voxels show bright (red marks) due to the in-flow effect and venule voxels show as dark dots (blue marks) because of the fast T_2_* decay (**Fig 2B**).

### bSSFP BOLD- and CBV-fMRI

The bSSFP sequence was applied to acquire the evoked BOLD signals by using the following parameters: TR = 15.6 ms; TE = 7.8 ms; flip angle = 15°; matrix = 96 × 96; FOV = 9.6 × 9.6 mm^2^; in-plane resolution = 100 × 100 μm^2^; slice thickness = 500 μm. For the bSSFP CBV-fMRI, the parameters were adjusted with TR = 10.4 ms and TE = 5.2 ms. The total TR to acquire each image is 1 second. To reach the steady state, 300 dummy scans were used, followed by 25 pre-stimulation scans, 1 scan during stimulation, and 44 post-stimulation scans with 10 epochs for each trial. The fMRI stimuli block design of each trial consisted of 10-second stimulation and 35-second interstimulus interval. The total acquisition duration of each trial was 7 minutes 55 seconds. CBV-weighted fMRI signals were acquired after intravenous injection of dextran-coated iron oxide particles (15 ~ 20 mg of Fe/kg, BioPAL).

### PC-MRI

To measure the flow velocity of individual arterioles and venules, the PC-MRI sequence was applied with the following parameters. For the in vitro phantom and in vivo single-vessel CBFv measurement: TR = 15.6 ms; TE = 4.2, 4.5, 5.0, 6.0 ms; flip angle = 25°; FOV = 6.4 × 6.4 mm^2^; matrix = 128 × 128; in-plane resolution = 50 × 50 μm^2^; slice thickness = 500 μm; maximum velocity encoding (Venc) = 0.66 to 1.56 cm/s (based on the flow values). The total acquisition time was 11 minutes 28 seconds. For the in vivo CBFv-fMRI measurements: TR = 15.6 ms; TE = 5 ms; flip angle = 30°; FOV = 6.4 × 6.4 mm^2^; matrix = 64 × 64; in-plane resolution = 100 × 100 μm^2^; slice thickness = 500 μm. A total TR for each image is 4 seconds. The total acquisition duration of each trial was 16 minutes. The total acquisition duration of each trial was 16 minutes. For in vivo single-vessel CBFv measurement with 9.4T scanner: TR = 15.6 ms; TE = 5.0 ms; flip angle = 15°; FOV = 6.4 × 6.4 mm^2^; matrix = 96 × 96; in-plane resolution = 67 × 67 μm^2^; slice thickness = 500 μm; maximum Venc = 0.91 to 1.58 cm/s (based on velocity values). To measure the blood flow velocity, bipolar flow encoding gradients were applied along the slice encoding direction. The slice position was anatomically identical with the slice position of the MGE imaging.

### Parameter optimization for the single-vessel CBFv mapping

(i) The orientation of the 2D slice should be parallel to the x-z plane of the magnet when performing slice-oriented velocity measurement, which would reduce cross-axis gradient-dependent error. (ii) The Venc value needed to be matched with the maximal velocities likely to be encountered within the vessel of interest. Since the Venc value is inversely proportional to the bipolar gradient, the minimal Venc value depends on the gradient strength. It should be noted that too low Venc values could also lead to gradient heating artifacts due to the high duty cycle. (iii) We usually applied high-order MapShim to improve the field homogeneity before the high-resolution PC-MRI. Meanwhile, to remove field homogeneity-related phase offset, a pair of toggled bipolar gradients was typically applied. It is important to note that the mismatched amplitudes of toggled bipolar gradient amplitudes or related eddy currents could lead to background velocity error. We could manually calibrate the appropriate gradient amplitude using the build-in parameters of the Bruker system, i.e., PVM_PPGradAmpArray.

### Data analysis and statistics

All data preprocessing and analysis were performed by using the software package, Analysis of Functional NeuroImages (AFNI) (NIH, Bethesda, USA). All relevant fMRI analysis source codes can be downloaded from (https://afni.nimh.nih.gov/pub/dist/doc/htmldoc/background_install/main_toc.html).

### Definition of the individual vessels

The individual arteriole/venule voxels were defined by the signal intensity of the A–V map [22]. The arterioles are determined if the voxel intensities are higher than the mean signal intensities plus 2 times the standard deviation of the local area in a 5 × 5 kernel. The venules are determined if the voxel intensities are lower than the mean signal intensities minus 2 times the standard deviation of the local area [22,38,39]. The locations of individual arteriole/venule voxels defined in the A–V map were used to extract the time courses of BOLD/CBV-fMRI for individual vessels.

### BOLD/CBV-fMRI and PC-MRI data analysis

To register the evoked bSSFP-fMRI images and evoked PC-MRI images with the 2D anatomical A–V map, the tag-based registration method was applied. A total of 12 to 15 tags were selected from the averaged bSSFP-fMRI images or the averaged PC-fMRI images to register those selected from the A–V map. We used a 3dLocalstat AFNI function to normalize the signal intensity of the single-vessel maps. This process allowed us to plot the PC-based velocity values of individual vessel voxels to the normalized signal intensity of A–V maps. For the evoked signals, the bSSFP-fMRI images and PC-MRI images were normalized by scaling the baseline to 100. Multiple trials of block design time courses were averaged for each animal. No additional smoothing step was applied. The β-value was calculated to measure the amplitude of the fMRI responses at each TR. The voxel-wise β map was illustrated with the spatial pattern of the fMRI responses corresponding to the different time points after the stimulus onset. After registration (tag-based registration) and region of interest extraction (3dLocalstat function, mask shown in **Fig 2B**), we extracted the PC-based flow velocity values from individual vessel voxels, which were identified based on the algorithm as described in the previous section.

The hemodynamic response function is based on the “block function” of 3dDeconvolve module developed in AFNI. The HRF model is defined as follows:

$$\text{h}(\text{t})=\int_{0}^{\min(t,L)}s^{4}\;e^{-s}/[4^{4}e^{-4}]\;\text{ds}$$

Gamma variate function = s^4^e^−s^ /4^4^e^−4^. L was the duration of the response. BLOCK (L, 1) is a convolution of a square wave of duration L that makes a peak amplitude of block response = 1.

For the resting-state single-vessel CBFv-fMRI correlation analysis, the seed-based voxel-wise correlation map was created using AFNI. The Welch power spectrum density plot was calculated from arteriole and venule voxels with averaged periodogram method (a 240-second timing window into overlapping sections with 60-point and 25% overlap). The sampling rate of rs-CBFv-fMRI signal is 4 seconds, allowing the analysis of low-frequency signal oscillation less than 0.125 Hz.

For the group analysis, Student *t* test was performed, and error bars are displayed as the means ± SEM. The *p*-values < 0.05 were considered statistically significant. The sample size of animal experiments is not previously estimated. No blinding and randomization design was needed in this work.

## Supporting information

S1 Fig The preparation of in vivo experiment for the PC-MRI in 14.1 T. **(A)** The flowchart of the in vivo experiment in the 14.1 T scanner. **(B)** Photograph of the custom-made transceiver surface RF coil. **(C)** Photograph of the coil position: The coil is glued to the rat skull. **(D)** The schematic drawing of the rat position inside the MRI holder. **(E)** Representative images from different views of the FLASH MRI show the ideal coil position. FLASH, fast low angle shot; PC, phase contrast; RF, radio frequency.
(TIFF)

S2 Fig Phase images from the different representative rats with different TEs and flip angles. **(A)** Phase images from a representative rat with different TEs, i.e., 2.75, 3.0, and 3.2 ms. The right panel shows the mean blood flow velocity (mean ± SEM) from left images with N_Arteriole_ = 48 and N_Venule_ = 22. **(B)** Phase images from a representative rat with different flip angles, i.e., 25°, 30°, and 35°. The right panel shows the mean CBFv from left images with N_Arteriole_ = 38 and N_Venule_ = 14. The data underlying this figure can be found in S4 Data. CBFv, cerebral blood flow–related velocity; TE, echo time.
(TIFF)

S3 Fig The bSSFP-based single-vessel BOLD/CBV-fMRI and the PC-based single-vessel CBFv measurement from a representative rat. **(A)** The evoked bSSFP-based BOLD- (left) and CBV- (right) fMRI maps overlaid on the A–V map of a representative rat, with the voxel-wise time courses from the ROIs of individual venule and arteriole voxels (10 seconds on and 35 seconds off for 10 epochs plotted in a 3 × 3 matrix). **(B)** The evoked CBFv functional maps overlaid on the A–V map of a representative rat. The voxel-wise time courses of CBFv changes from the same ROIs of individual venule and arteriole voxels (10 seconds on and 50 seconds off for 12 epochs plotted in a 3 x 3 matrix). A–V, arteriole–venule; BOLD, blood oxygenation level–dependent; bSSFP, balanced steady-state free precession; CBFv, cerebral blood flow–related velocity; CBV, cerebral blood volume; PC, phase contrast; ROI, region of interest.
(TIFF)

S4 Fig The PC-based single-vessel resting-state CBFv-fMRI mapping. **(A)** The A–V map shows bright dot as arterioles and dark dots as venules from one representative rat. Seed-based correlation maps were overlapped on the A–V map, showing 2 venule seeds (V1 and V2) and 2 arteriole seeds (A1 and A2). The venule-based correlation map shows positive correlations to other venules, but negative correlations with arterioles. In contrast, the arteriole-based correlation map shows positive correlations to other arterioles, but negative correlations with venules. **(B)** The enlarged CBFv map shows the venule with positive velocity (bright dots) and arterioles with negative velocity (dark dots). The venule seed-based CBFv correlation map was overlapped on the CBFv map, showing the positive correlation on surrounding venule voxels (bright dots), and negative correlation on surrounding arteriole voxels (dark dots). **(C)** The normalized time course extracted from venule and arteriole voxels, showing correlated low-frequency signal fluctuation. **(D)** The PSD plot of resting state CBFv dynamics from arteriole and venule voxels shows the slightly higher power from 0.01 to 0.04Hz (*n* = 5). The data underlying this figure can be found in S5 Data. A–V, arteriole–venule; CBFv, cerebral blood flow–related velocity; PC, phase contrast; PSD, power spectrum density.
(TIFF)

S5 Fig The PC-based CBFv mapping in rats with the 9.4 T scanner. **(A)** The vectorized flow velocity (CBFv) map with 67 × 67 um^2^ in-plane resolution shows venules (bright dots) with positive CBFv values and arterioles (dark dots) with negative CBFv values. **(B)** The bar graph shows the averaged velocity for arterioles (−1.37 ± 0.94 mm/s) and venules (1.02 ± 0.32 mm/s) from 4 rats. The data underlying this figure can be found in S6 Data. CBFv, cerebral blood flow–related velocity; PC, phase contrast.
(TIFF)

S1 Movie The bSSFP-based single-vessel BOLD/CBV-fMRI and the PC-MRI based single-vessel CBFv-fMRI in the rat cortex. The left panel shows the evoked bSSFP-based BOLD- (upper) and CBV- (lower) fMRI responses with a representative trace from single venule and arteriole voxels (arrow) (10 seconds on and 35 seconds off for a total of 45-second time window with TR = 1 second). The right panel shows the evoked PC-based CBFv-fMRI responses with a representative trace from single venule (upper) and arteriole (lower) voxels (10 seconds on and 50 seconds off for a total of 60-second time window with TR = 4 seconds). Note that the underlay is the A–V map showing venules as dark voxels and arterioles as bright voxels. A–V, arteriole–venule; BOLD, blood oxygenation level–dependent; bSSFP, balanced steady-state free precession; CBFv, cerebral blood flow–related velocity; CBV, cerebral blood volume; PC, phase contrast.
(MP4)

S1 Data The flow velocity estimates from the 5 ROIs with different TEs and different pump rates. The flow velocity estimates from the 5 ROIs with different TEs (4.2, 4.5, 5.0, and 6.0 ms) and different pump rates (0, 2.3, 4.5, 6.8, and 9 mm/s). The linear fitting is calculated from averaged velocity of venules and arterioles.ROI, region of interest; TE, echo time.
(XLSX)

S2 Data Scatter plot of the flow velocities from individual arterioles and venules as the function of the normalized signal intensities of each vessel in the A–V map from 11 rats. **Fig 2D upper panel:** The flow velocities from individual arterioles and venules as the function of the normalized signal intensities of each vessel in the A–V map. The raw data was acquired from 11 animals. **Fig 2D lower panel:** Mean velocity of arterioles and venules for the bar graph from 11 animals. **MATLAB code and mat file:** The MATLAB code and raw data for the histogram of the blood velocity distribution across arterioles and venules from the animals. A–V, arteriole–venule.
(RAR)

S3 Data Hemodynamic signal data for single-vessel BOLD, CBV, and CBFv-fMRI measurement. **Fig 3B:** The time courses of evoked bSSFP-BOLD and CBV-fMRI with the block design stimulation paradigm from venules and arterioles. **Fig 3C:** The averaged time courses of the fractional change for evoked BOLD and CBV signals from venule and arteriole ROIs and the raw data from 3 animals. **Fig 3D:** The time courses of the evoked CBFv changes from the arteriole and venule ROIs from both arterioles and venules with the block design stimulation paradigm. **Fig 3E:** The averaged time courses of the evoked CBFv changes from arteriole and venule ROIs with the block design stimulation paradigm and the raw data from 5 animals. **Fig 3F:** The venules and arterioles ROIs peak signals for BOLD, CBV, and CBFv measurements from different animals. BOLD, blood oxygenation level–dependent; bSSFP, balanced steady-state free precession; CBFv, cerebral blood flow–related velocity; CBV, cerebral blood volume; ROI, region of interest.
(XLSX)

S4 Data Blood flow velocity with different TEs and flip angles. **S2A Fig:** The blood flow velocity of 48 arterioles and 22 venules with different TEs (2.75, 3.0, and 3.2 ms). **S2B Fig:** The blood flow velocity of 38 arterioles and 14 venules with different flip angles (25°, 30°, 35°). TE, echo time.
(XLSX)

S5 Data The PC-based single-vessel resting-state CBFv-fMRI PSD. The PSD of resting state CBFv dynamics from arteriole and venule voxels from 5 animals. CBFv, cerebral blood flow–related velocity; PC, phase contrast; PSD, power spectrum density.
(XLSX)

S6 Data The PC-based CBFv mapping in rats with the 9.4 T scanner. The averaged velocity for arterioles and venules from 4 animals. CBFv, cerebral blood flow–related velocity; PC, phase contrast.
(XLSX)

## Abbreviations

AD: Alzheimer disease
AFNI: Analysis of Functional NeuroImages
ASL: arterial spin labeling
A–V: arteriole–venule
BOLD: blood oxygenation level–dependent
bSSFP: balanced steady-state free precession
CBF: cerebral blood flow
CBFv: cerebral blood flow–related velocity
CBV: cerebral blood volume
CNR: contrast-to-noise ratio
fMRI: functional MRI
MGE: multi-gradient echo
PC: phase contrast
RF: radio frequency
SNR: signal-to-noise ratio
TE: echo time
TR: repetition time
VASO: vascular-space-occupancy
Venc: velocity encoding

## References

[1] Attwell D, Buchan AM, Charpak S, Lauritzen M, MacVicar BA, Newman EA. Glial and neuronal control of brain blood flow. Nature. 2010;468(7321):232–43. doi: 10.1038/nature09613 21068832 PMC3206737

[2] Shih AY, Driscoll JD, Drew PJ, Nishimura N, Schaffer CB, Kleinfeld D. Two-photon microscopy as a tool to study blood flow and neurovascular coupling in the rodent brain. J Cereb Blood Flow Metab. 2012;32(7):1277–309. doi: 10.1038/jcbfm.2011.196 ; PubMed Central PMCID: PMC3390800. 22293983 PMC3390800

[3] Kleinfeld D, Mitra PP, Helmchen F, Denk W. Fluctuations and stimulus-induced changes in blood flow observed in individual capillaries in layers 2 through 4 of rat neocortex. Proc Natl Acad Sci U S A. 1998;95(26):15741–6. doi: 10.1073/pnas.95.26.15741 ; PubMed Central PMCID: PMC28114. 9861040 PMC28114

[4] Srinivasan VJ, Sakadzic S, Gorczynska I, Ruvinskaya S, Wu W, Fujimoto JG, et al. Depth-resolved microscopy of cortical hemodynamics with optical coherence tomography. Opt Lett. 2009;34(20):3086–8. doi: 10.1364/OL.34.003086 ; PubMed Central PMCID: PMC2778254. 19838234 PMC2778254

[5] Yao J, Maslov KI, Shi Y, Taber LA, Wang LV. In vivo photoacoustic imaging of transverse blood flow by using Doppler broadening of bandwidth. Opt Lett. 2010;35(9):1419–21. doi: 10.1364/OL.35.001419 ; PubMed Central PMCID: PMC2916025. 20436589 PMC2916025

[6] Ances BM, Greenberg JH, Detre JA. Laser doppler imaging of activation-flow coupling in the rat somatosensory cortex. NeuroImage. 1999;10(6):716–23. doi: 10.1006/nimg.1999.0510 . 10600417

[7] Dunn AK, Bolay H, Moskowitz MA, Boas DA. Dynamic imaging of cerebral blood flow using laser speckle. J Cereb Blood Flow Metab. 2001;21(3):195–201. doi: 10.1097/00004647-200103000-00002 . 11295873

[8] Mace E, Montaldo G, Cohen I, Baulac M, Fink M, Tanter M. Functional ultrasound imaging of the brain. Nat Methods. 2011;8(8):662–4. doi: 10.1038/nmeth.1641 . 21725300

[9] Sieu LA, Bergel A, Tiran E, Deffieux T, Pernot M, Gennisson JL, et al. EEG and functional ultrasound imaging in mobile rats. Nat Methods. 2015;12(9):831–4. Epub 2015/08/04. doi: 10.1038/nmeth.3506 ; PubMed Central PMCID: PMC4671306. 26237228 PMC4671306

[10] Rungta RL, Osmanski BF, Boido D, Tanter M, Charpak S. Light controls cerebral blood flow in naive animals. Nat Commun. 2017;8:14191. Epub 2017/02/01. doi: 10.1038/ncomms14191; PubMed Central PMCID: PMC5290324. 28139643 PMC5290324

[11] Wang T, Wu C, Ouzounov DG, Gu W, Xia F, Kim M, et al. Quantitative analysis of 1300-nm three-photon calcium imaging in the mouse brain. Elife. 2020;9. Epub 2020/01/31. doi: 10.7554/eLife.53205; PubMed Central PMCID: PMC7028383. 31999253 PMC7028383

[12] Xie Y, Chan AW, McGirr A, Xue S, Xiao D, Zeng H, et al. Resolution of High-Frequency Mesoscale Intracortical Maps Using the Genetically Encoded Glutamate Sensor iGluSnFR. J Neurosci. 2016;36(4):1261–72. Epub 2016/01/29. doi: 10.1523/JNEUROSCI.2744-15.2016 ; PubMed Central PMCID: PMC6604822. 26818514 PMC6604822

[13] Ovsepian SV, Jiang Y, Sardella TCP, Malekzadeh-Najafabadi J, Burton NC, Yu X, et al. Visualizing cortical response to optogenetic stimulation and sensory inputs using multispectral handheld optoacoustic imaging. Photodermatology. 2020;17:100153. Epub 2020/03/11. doi: 10.1016/j.pacs.2019.100153; PubMed Central PMCID: PMC7052434. 32154103 PMC7052434

[14] Kwong KK, Belliveau JW, Chesler DA, Goldberg IE, Weisskoff RM, Poncelet BP, et al. Dynamic magnetic resonance imaging of human brain activity during primary sensory stimulation. Proc Natl Acad Sci U S A. 1992;89(12):5675–9. doi: 10.1073/pnas.89.12.5675 ; PubMed Central PMCID: PMC49355. 1608978 PMC49355

[15] Detre JA, Leigh JS, Williams DS, Koretsky AP. Perfusion imaging. Magn Reson Med. 1992;23(1):37–45. doi: 10.1002/mrm.1910230106 . 1734182

[16] Williams DS, Detre JA, Leigh JS, Koretsky AP. Magnetic resonance imaging of perfusion using spin inversion of arterial water. Proc Natl Acad Sci U S A. 1992;89(1):212–6. Epub 1992/01/01. doi: 10.1073/pnas.89.1.212 ; PubMed Central PMCID: PMC48206. 1729691 PMC48206

[17] Detre JA, Wang J. Technical aspects and utility of fMRI using BOLD and ASL. Clin Neurophysiol. 2002;113(5):621–34. Epub 2002/04/27. doi: 10.1016/s1388-2457(02)00038-x . 11976042

[18] Ogawa S, Lee TM, Kay AR, Tank DW. Brain magnetic resonance imaging with contrast dependent on blood oxygenation. Proc Natl Acad Sci U S A. 1990;87(24):9868–72. doi: 10.1073/pnas.87.24.9868 ; PubMed Central PMCID: PMC55275. 2124706 PMC55275

[19] Ogawa S, Tank DW, Menon R, Ellermann JM, Kim SG, Merkle H, et al. Intrinsic signal changes accompanying sensory stimulation: functional brain mapping with magnetic resonance imaging. Proc Natl Acad Sci U S A. 1992;89(13):5951–5. doi: 10.1073/pnas.89.13.5951 ; PubMed Central PMCID: PMC402116. 1631079 PMC402116

[20] Belliveau JW, Kennedy DN Jr., McKinstry RC, Buchbinder BR, Weisskoff RM, Cohen MS, et al. Functional mapping of the human visual cortex by magnetic resonance imaging. Science. 1991;254(5032):716–9. doi: 10.1126/science.1948051 . 1948051

[21] Lu H, Golay X, Pekar JJ, Van Zijl PC. Functional magnetic resonance imaging based on changes in vascular space occupancy. Magn Reson Med. 2003;50(2):263–74. Epub 2003/07/24. doi: 10.1002/mrm.10519 . 12876702

[22] Yu X, He Y, Wang M, Merkle H, Dodd SJ, Silva AC, et al. Sensory and optogenetically driven single-vessel fMRI. Nat Methods. 2016;13(4):337–40. doi: 10.1038/nmeth.3765 . 26855362 PMC6298439

[23] Huber L, Handwerker DA, Jangraw DC, Chen G, Hall A, Stuber C, et al. High-Resolution CBV-fMRI Allows Mapping of Laminar Activity and Connectivity of Cortical Input and Output in Human M1. Neuron. 2017;96(6):1253–63 e7. Epub 2017/12/12. doi: 10.1016/j.neuron.2017.11.005 ; PubMed Central PMCID: PMC5739950. 29224727 PMC5739950

[24] Park SH, Do WJ, Choi SH, Zhao T, Bae KT. Mapping blood flow directionality in the human brain. Magn Reson Imaging. 2016;34(6):754–64. Epub 2016/03/13. doi: 10.1016/j.mri.2016.03.005 . 26968145

[25] Frank LR, Lu K, Wong EC. Perfusion tensor imaging. Magn Reson Med. 2008;60(6):1284–91. Epub 2008/11/26. doi: 10.1002/mrm.21806 ; PubMed Central PMCID: PMC4319714. 19030161 PMC4319714

[26] Wong EC, Cronin M, Wu WC, Inglis B, Frank LR, Liu TT. Velocity-selective arterial spin labeling. Magn Reson Med. 2006;55(6):1334–41. Epub 2006/05/16. doi: 10.1002/mrm.20906 . 16700025

[27] Wu WC, Wong EC. Feasibility of velocity selective arterial spin labeling in functional MRI. J Cereb Blood Flow Metab. 2007;27(4):831–8. Epub 2006/08/24. doi: 10.1038/sj.jcbfm.9600386 . 16926843

[28] Moran PR. A flow velocity zeugmatographic interlace for NMR imaging in humans. Magn Reson Imaging. 1982;1(4):197–203. Epub 1982/01/01. doi: 10.1016/0730-725x(82)90170-9 . 6927206

[29] Bryant DJ, Payne JA, Firmin DN, Longmore DB. Measurement of flow with NMR imaging using a gradient pulse and phase difference technique. J Comput Assist Tomogr. 1984;8(4):588–93. Epub 1984/08/01. doi: 10.1097/00004728-198408000-00002 . 6736356

[30] Zong X, Lin W. Quantitative phase contrast MRI of penetrating arteries in centrum semiovale at 7T. NeuroImage. 2019;195:463–74. Epub 2019/04/03. doi: 10.1016/j.neuroimage.2019.03.059 . 30935910

[31] Bouvy WH, Geurts LJ, Kuijf HJ, Luijten PR, Kappelle LJ, Biessels GJ, et al. Assessment of blood flow velocity and pulsatility in cerebral perforating arteries with 7-T quantitative flow MRI. NMR Biomed. 2016;29(9):1295–304. Epub 2015/04/29. doi: 10.1002/nbm.3306 ; PubMed Central PMCID: PMC5008170. 25916399 PMC5008170

[32] Kang CK, Park CA, Lee DS, Lee YB, Park CW, Kim YB, et al. Velocity measurement of microvessels using phase-contrast magnetic resonance angiography at 7 Tesla MRI. Magn Reson Med. 2016;75(4):1640–6. Epub 2015/05/20. doi: 10.1002/mrm.25600 . 25980462

[33] Schnerr RS, Jansen JFA, Uludag K, Hofman PAM, Wildberger JE, van Oostenbrugge RJ, et al. Pulsatility of Lenticulostriate Arteries Assessed by 7 Tesla Flow MRI-Measurement, Reproducibility, and Applicability to Aging Effect. Front Physiol. 2017;8:961. Epub 2017/12/12. doi: 10.3389/fphys.2017.00961; PubMed Central PMCID: PMC5705621. 29225580 PMC5705621

[34] Wei Z, Chen L, Lin Z, Jiang D, Xu J, Liu P, et al. Optimization of phase-contrast MRI for the estimation of global cerebral blood flow of mice at 11.7T. Magn Reson Med. 2019;81(4):2566–75. Epub 2018/11/06. doi: 10.1002/mrm.27592 ; PubMed Central PMCID: PMC6372341. 30393888 PMC6372341

[35] Dolui S, Wang Z, Wang DJJ, Mattay R, Finkel M, Elliott M, et al. Comparison of non-invasive MRI measurements of cerebral blood flow in a large multisite cohort. J Cereb Blood Flow Metab. 2016;36(7):1244–56. Epub 2016/05/05. doi: 10.1177/0271678X16646124 ; PubMed Central PMCID: PMC4929707. 27142868 PMC4929707

[36] Geurts LJ, Bhogal AA, Siero JCW, Luijten PR, Biessels GJ, Zwanenburg JJM. Vascular reactivity in small cerebral perforating arteries with 7T phase contrast MRI—A proof of concept study. NeuroImage. 2018;172:470–7. Epub 2018/02/07. doi: 10.1016/j.neuroimage.2018.01.055 ; PubMed Central PMCID: PMC5915583. 29408324 PMC5915583

[37] Hoogeveen RM, Bakker CJ, Viergever MA. MR phase-contrast flow measurement with limited spatial resolution in small vessels: value of model-based image analysis. Magn Reson Med. 1999;41(3):520–8. Epub 1999/04/16. doi: 10.1002/(sici)1522-2594(199903)41:3<520::aid-mrm14>3.0.co;2-a . 10204875

[38] He Y, Wang M, Chen X, Pohmann R, Polimeni JR, Scheffler K, et al. Ultra-Slow Single-Vessel BOLD and CBV-Based fMRI Spatiotemporal Dynamics and Their Correlation with Neuronal Intracellular Calcium Signals. Neuron. 2018;97(4):925–39 e5. Epub 2018/02/06. doi: 10.1016/j.neuron.2018.01.025 ; PubMed Central PMCID: PMC5845844. 29398359 PMC5845844

[39] Chen X, Sobczak F, Chen Y, Jiang Y, Qian C, Lu Z, et al. Mapping optogenetically-driven single-vessel fMRI with concurrent neuronal calcium recordings in the rat hippocampus. Nat Commun. 2019;10(1):5239. doi: 10.1038/s41467-019-12850-x; PubMed Central PMCID: PMC6868210. 31748553 PMC6868210

[40] Walker PG, Cranney GB, Scheidegger MB, Waseleski G, Pohost GM, Yoganathan AP. Semiautomated method for noise reduction and background phase error correction in MR phase velocity data. J Magn Reson Imaging. 1993;3(3):521–30. Epub 1993/05/01. doi: 10.1002/jmri.1880030315 . 8324312

[41] Peeters JM, Bos C, Bakker CJ. Analysis and correction of gradient nonlinearity and B0 inhomogeneity related scaling errors in two-dimensional phase contrast flow measurements. Magn Reson Med. 2005;53(1):126–33. Epub 2005/02/04. doi: 10.1002/mrm.20309 . 15690511

[42] Bernstein MA, Zhou XJ, Polzin JA, King KF, Ganin A, Pelc NJ, et al. Concomitant gradient terms in phase contrast MR: analysis and correction. Magn Reson Med. 1998;39(2):300–8. Epub 1998/02/20. doi: 10.1002/mrm.1910390218 . 9469714

[43] Schaffer CB, Friedman B, Nishimura N, Schroeder LF, Tsai PS, Ebner FF, et al. Two-photon imaging of cortical surface microvessels reveals a robust redistribution in blood flow after vascular occlusion. PLoS Biol. 2006;4(2):e22. Epub 2005/12/29. doi: 10.1371/journal.pbio.0040022; PubMed Central PMCID: PMC1324794. 16379497 PMC1324794

[44] Shih AY, Blinder P, Tsai PS, Friedman B, Stanley G, Lyden PD, et al. The smallest stroke: occlusion of one penetrating vessel leads to infarction and a cognitive deficit. Nat Neurosci. 2013;16(1):55–63. Epub 2012/12/18. doi: 10.1038/nn.3278 ; PubMed Central PMCID: PMC3952571. 23242312 PMC3952571

[45] Silva AC, Koretsky AP, Duyn JH. Functional MRI impulse response for BOLD and CBV contrast in rat somatosensory cortex. Magn Reson Med. 2007;57(6):1110–8. doi: 10.1002/mrm.21246 PubMed PMID: WOS:000246979900015. 17534912 PMC4756432

[46] Drew PJ, Shih AY, Kleinfeld D. Fluctuating and sensory-induced vasodynamics in rodent cortex extend arteriole capacity. Proc Natl Acad Sci U S A. 2011;108(20):8473–8. doi: 10.1073/pnas.1100428108 ; PubMed Central PMCID: PMC3100929. 21536897 PMC3100929

[47] Rungta RL, Chaigneau E, Osmanski BF, Charpak S. Vascular Compartmentalization of Functional Hyperemia from the Synapse to the Pia. Neuron. 2018;99(2):362–75 e4. Epub 2018/06/26. doi: 10.1016/j.neuron.2018.06.012 ; PubMed Central PMCID: PMC6069674. 29937277 PMC6069674

[48] Koretsky AP. Early development of arterial spin labeling to measure regional brain blood flow by MRI. NeuroImage. 2012;62(2):602–7. Epub 2012/01/17. doi: 10.1016/j.neuroimage.2012.01.005 ; PubMed Central PMCID: PMC4199083. 22245338 PMC4199083

[49] Wang J, Fernandez-Seara MA, Wang S, St Lawrence KS. When perfusion meets diffusion: in vivo measurement of water permeability in human brain. J Cereb Blood Flow Metab. 2007;27(4):839–49. Epub 2006/09/14. doi: 10.1038/sj.jcbfm.9600398 . 16969383

[50] Hutchinson EB, Stefanovic B, Koretsky AP, Silva AC. Spatial flow-volume dissociation of the cerebral microcirculatory response to mild hypercapnia. NeuroImage. 2006;32(2):520–30. doi: 10.1016/j.neuroimage.2006.03.033 . 16713717

[51] Kobat D, Durst ME, Nishimura N, Wong AW, Schaffer CB, Xu C. Deep tissue multiphoton microscopy using longer wavelength excitation. Opt Express. 2009;17(16):13354–64. doi: 10.1364/oe.17.013354 19654740

[52] Kobat D, Horton NG, Xu C. In vivo two-photon microscopy to 1.6-mm depth in mouse cortex. J Biomed Opt. 2011;16(10):106014. Epub 2011/10/28. doi: 10.1117/1.3646209. 22029361

[53] Drew PJ, Shih AY, Driscoll JD, Knutsen PM, Blinder P, Davalos D, et al. Chronic optical access through a polished and reinforced thinned skull. Nat Methods. 2010;7(12):981–4. Epub 2010/10/23. doi: 10.1038/nmeth.1530 ; PubMed Central PMCID: PMC3204312. 20966916 PMC3204312

[54] Xu HT, Pan F, Yang G, Gan WB. Choice of cranial window type for in vivo imaging affects dendritic spine turnover in the cortex. Nat Neurosci. 2007;10(5):549–51. Epub 2007/04/10. doi: 10.1038/nn1883 . 17417634

[55] Dorand RD, Barkauskas DS, Evans TA, Petrosiute A, Huang AY. Comparison of intravital thinned skull and cranial window approaches to study CNS immunobiology in the mouse cortex. Intravital. 2014;3(2). Epub 2015/01/09. doi: 10.4161/intv.29728; PubMed Central PMCID: PMC4283137. 25568834 PMC4283137

[56] Cole JT, Yarnell A, Kean WS, Gold E, Lewis B, Ren M, et al. Craniotomy: true sham for traumatic brain injury, or a sham of a sham? J Neurotrauma. 2011;28(3):359–69. Epub 2010/12/31. doi: 10.1089/neu.2010.1427 ; PubMed Central PMCID: PMC3057208. 21190398 PMC3057208

[57] Davis TL, Kwong KK, Weisskoff RM, Rosen BR. Calibrated functional MRI: mapping the dynamics of oxidative metabolism. Proc Natl Acad Sci U S A. 1998;95(4):1834–9. Epub 1998/03/21. doi: 10.1073/pnas.95.4.1834 ; PubMed Central PMCID: PMC19199. 9465103 PMC19199

[58] Leontiev O, Dubowitz DJ, Buxton RB. CBF/CMRO2 coupling measured with calibrated BOLD fMRI: sources of bias. NeuroImage. 2007;36(4):1110–22. Epub 2007/05/26. doi: 10.1016/j.neuroimage.2006.12.034 ; PubMed Central PMCID: PMC2038985. 17524665 PMC2038985

[59] Mateo C, Knutsen PM, Tsai PS, Shih AY, Kleinfeld D. Entrainment of Arteriole Vasomotor Fluctuations by Neural Activity Is a Basis of Blood-Oxygenation-Level-Dependent "Resting-State" Connectivity. Neuron. 2017;96(4):936–48 e3. doi: 10.1016/j.neuron.2017.10.012 ; PubMed Central PMCID: PMC5851777. 29107517 PMC5851777

[60] van Veluw SJ, Hou SS, Calvo-Rodriguez M, Arbel-Ornath M, Snyder AC, Frosch MP, et al. Vasomotion as a Driving Force for Paravascular Clearance in the Awake Mouse Brain. Neuron. 2020;105(3):549–61 e5. Epub 2019/12/08. doi: 10.1016/j.neuron.2019.10.033 ; PubMed Central PMCID: PMC7028316. 31810839 PMC7028316

[61] Wagshul ME, Eide PK, Madsen JR. The pulsating brain: A review of experimental and clinical studies of intracranial pulsatility. Fluids Barriers CNS. 2011;8(1):5. Epub 2011/02/26. doi: 10.1186/2045-8118-8-5; PubMed Central PMCID: PMC3042979. 21349153 PMC3042979

[62] Driver ID, Traat M, Fasano F, Wise RG. Most Small Cerebral Cortical Veins Demonstrate Significant Flow Pulsatility: A Human Phase Contrast MRI Study at 7T. Front Neurosci. 2020;14:415. Epub 2020/05/21. doi: 10.3389/fnins.2020.00415; PubMed Central PMCID: PMC7214844. 32431591 PMC7214844

