# Correction: Assessment of single-vessel cerebral blood velocity by phase contrast fMRI

**DOI:** 10.1371/journal.pbio.3001951

**Published:** 2022-12-28

**Authors:** Xuming Chen, Yuanyuan Jiang, Sangcheon Choi, Rolf Pohmann, Klaus Scheffler, David Kleinfeld, Xin Yu

The affiliation for the seventh author is incomplete. Xin Yu is affiliated with:

Translational Neuroimaging and Neural Control Laboratory, Max Planck Institute for Biological Cybernetics, Tübingen, Germany

Current address: Athinoula A. Martinos Center for Biomedical Imaging, Massachusetts General Hospital and Harvard Medical School, Charlestown, Massachusetts, United States of America

## References

[1] Chen X, Jiang Y, Choi S, Pohmann R, Scheffler K, Kleinfeld D, et al. (2021) Assessment of single-vessel cerebral blood velocity by phase contrast fMRI. PLoS Biol 19(9): e3000923. 10.1371/journal.pbio.3000923 34499636 PMC8454982

