# Supplementary figures and images for: Assessment of single-vessel cerebral blood velocity by phase contrast fMRI
Source: PLoS Biol. 2021 Sep 9;19(9):e3000923. doi: 10.1371/journal.pbio.3000923 (PMC8454982; doi:10.1371/journal.pbio.3000923)

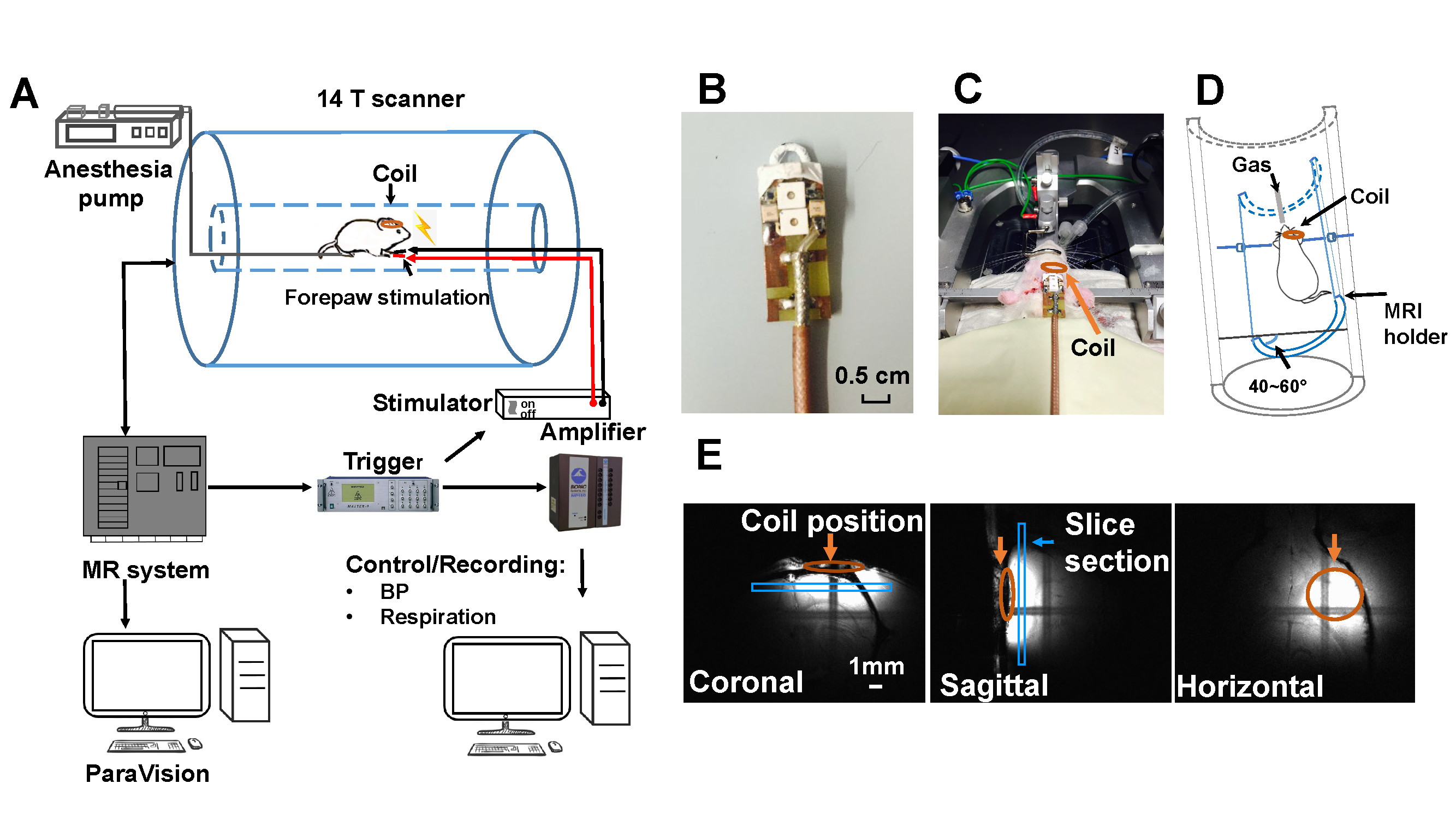

Supplement: S1 Fig — (A) The flowchart of the in vivo experiment in the 14.1 T scanner. (B) Photograph of the custom-made transceiver surface RF coil. (C) Photograph of the coil position: The coil is glued to the rat skull. (D) The schematic drawing of the rat position inside the MRI holder. (E) Representative images from different views of the FLASH MRI show the ideal coil position. FLASH, fast low angle shot; PC, phase contrast; RF, radio frequency. (TIFF) [file pbio.3000923.s001.tiff]

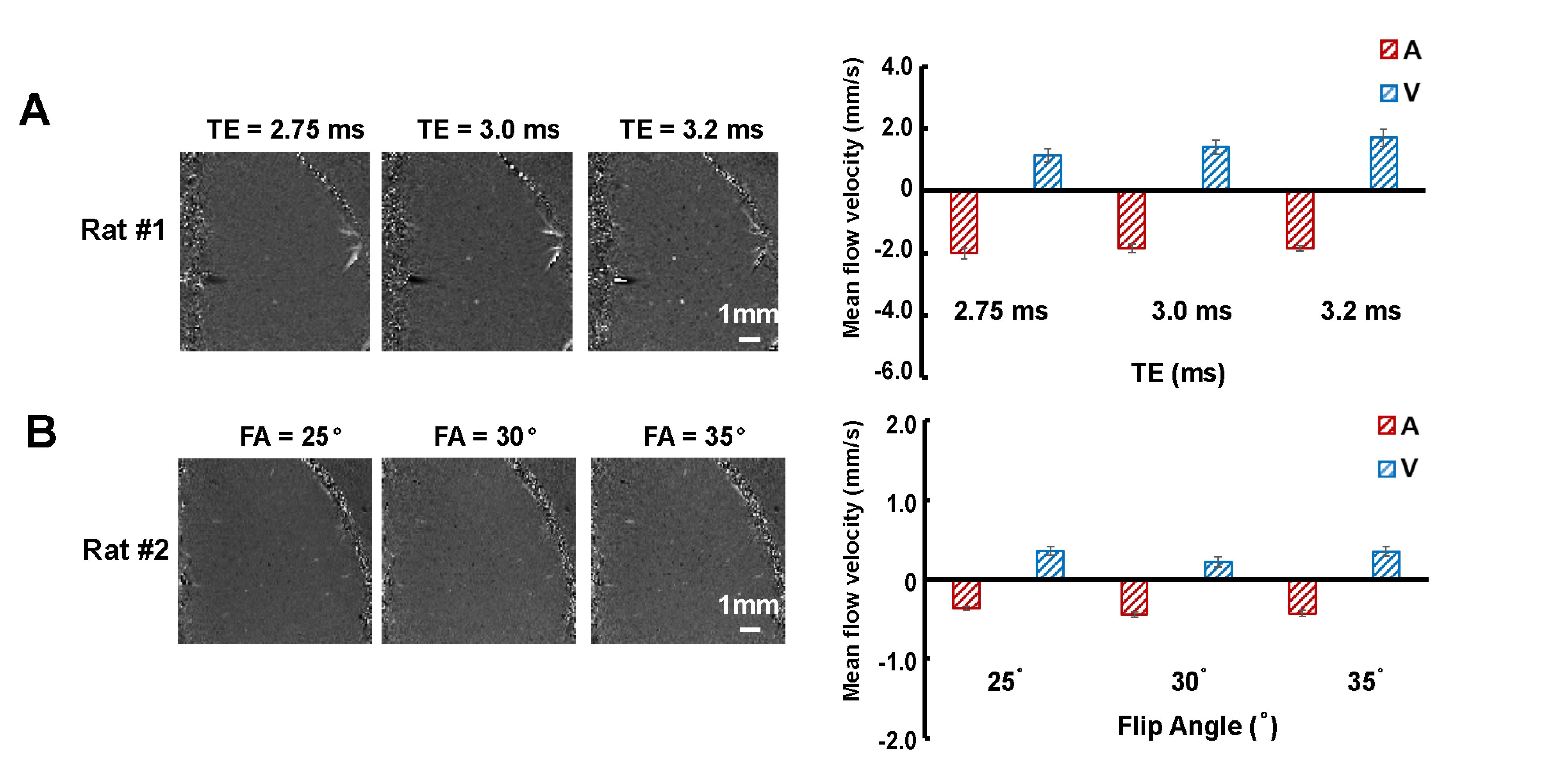

Supplement: S2 Fig — (A) Phase images from a representative rat with different TEs, i.e., 2.75, 3.0, and 3.2 ms. The right panel shows the mean blood flow velocity (mean ± SEM) from left images with NArteriole = 48 and NVenule = 22. (B) Phase images from a representative rat with different flip angles, i.e., 25°, 30°, and 35°. The right panel shows the mean CBFv from left images with NArteriole = 38 and NVenule = 14. The data underlying this figure can be found in S4 Data. CBFv, cerebral blood flow–related velocity; TE, echo time. (TIFF) [file pbio.3000923.s002.tiff]

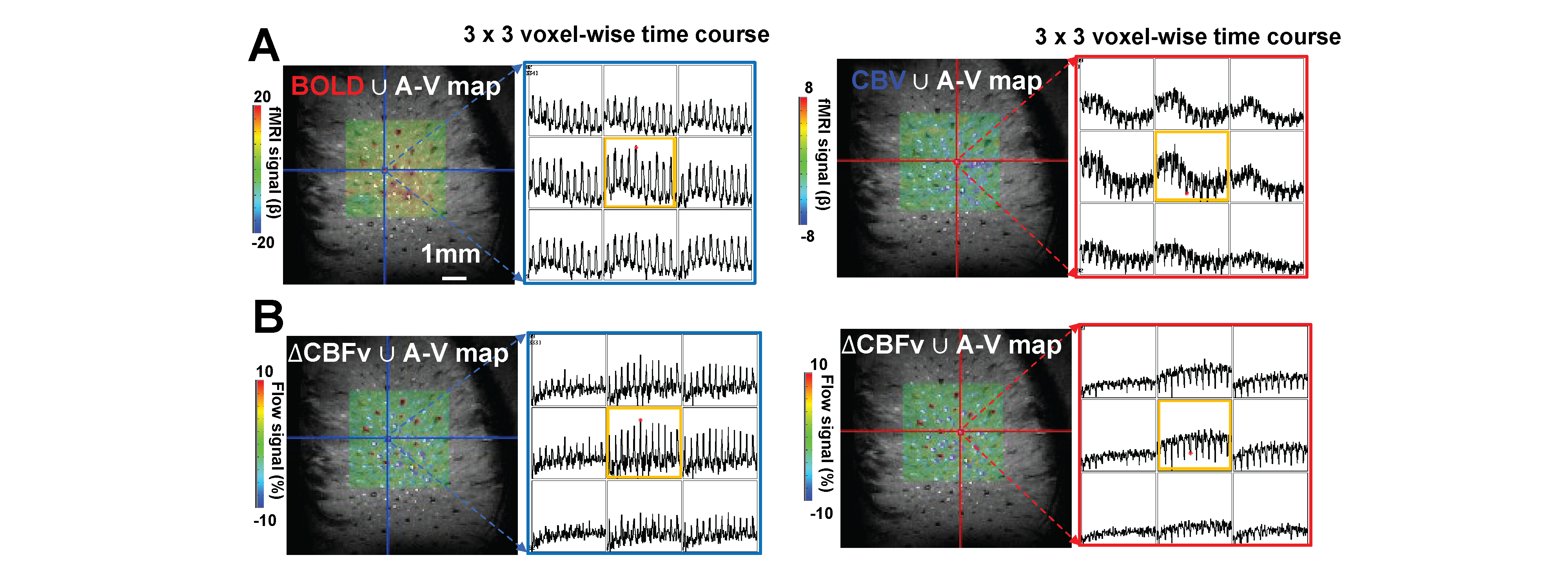

Supplement: S3 Fig — (A) The evoked bSSFP-based BOLD- (left) and CBV- (right) fMRI maps overlaid on the A–V map of a representative rat, with the voxel-wise time courses from the ROIs of individual venule and arteriole voxels (10 seconds on and 35 seconds off for 10 epochs plotted in a 3 × 3 matrix). (B) The evoked CBFv functional maps overlaid on the A–V map of a representative rat. The voxel-wise time courses of CBFv changes from the same ROIs of individual venule and arteriole voxels (10 seconds on and 50 seconds off for 12 epochs plotted in a 3 x 3 matrix). A–V, arteriole–venule; BOLD, blood oxygenation level–dependent; bSSFP, balanced steady-state free precession; CBFv, cerebral blood flow–related velocity; CBV, cerebral blood volume; PC, phase contrast; ROI, region of interest. (TIFF) [file pbio.3000923.s003.tiff]

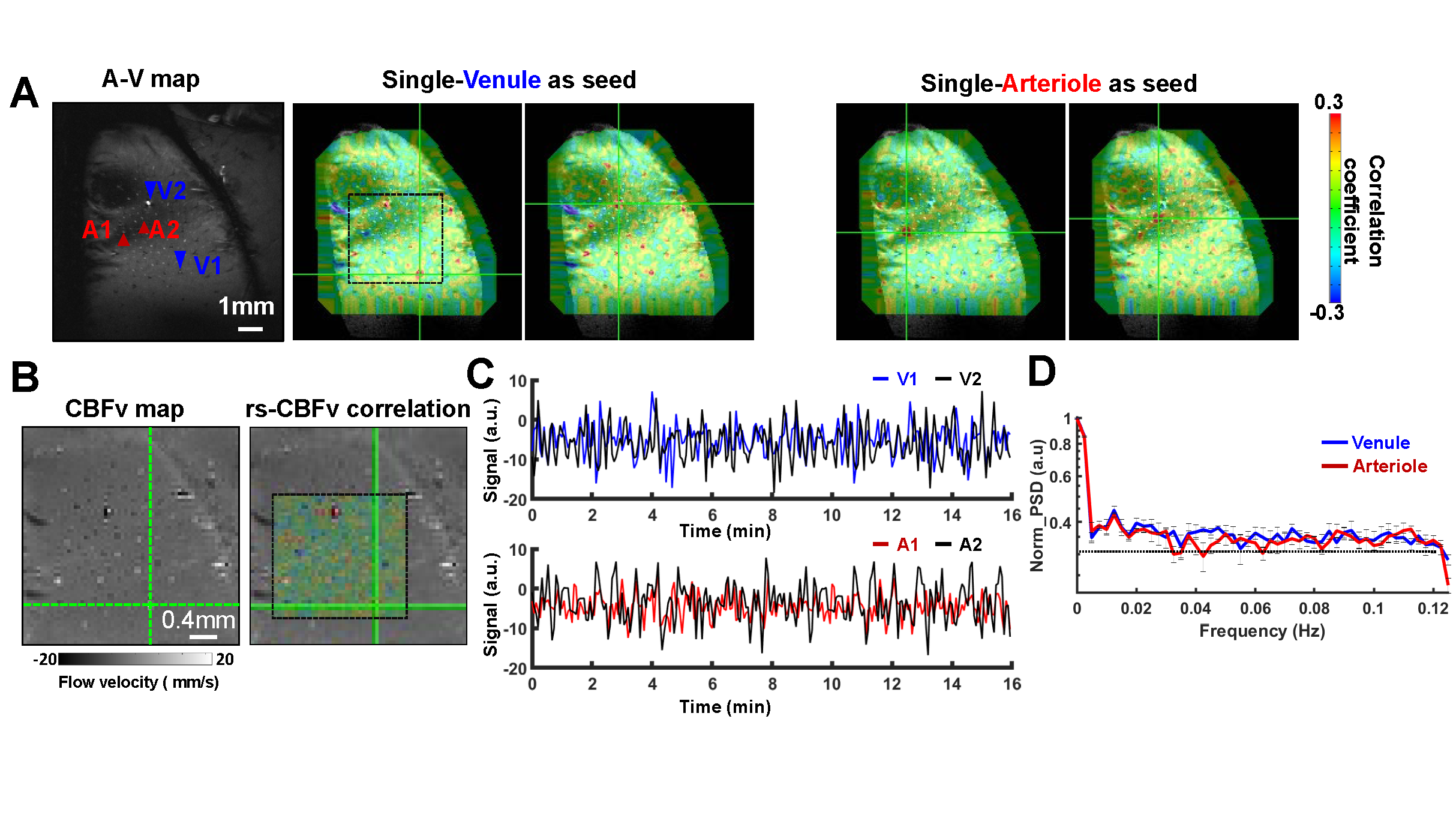

Supplement: S4 Fig — (A) The A–V map shows bright dot as arterioles and dark dots as venules from one representative rat. Seed-based correlation maps were overlapped on the A–V map, showing 2 venule seeds (V1 and V2) and 2 arteriole seeds (A1 and A2). The venule-based correlation map shows positive correlations to other venules, but negative correlations with arterioles. In contrast, the arteriole-based correlation map shows positive correlations to other arterioles, but negative correlations with venules. (B) The enlarged CBFv map shows the venule with positive velocity (bright dots) and arterioles with negative velocity (dark dots). The venule seed-based CBFv correlation map was overlapped on the CBFv map, showing the positive correlation on surrounding venule voxels (bright dots), and negative correlation on surrounding arteriole voxels (dark dots). (C) The normalized time course extracted from venule and arteriole voxels, showing correlated low-frequency signal fluctuation. (D) The PSD plot of resting state CBFv dynamics from arteriole and venule voxels shows the slightly higher power from 0.01 to 0.04Hz (n = 5). The data underlying this figure can be found in S5 Data. A–V, arteriole–venule; CBFv, cerebral blood flow–related velocity; PC, phase contrast; PSD, power spectrum density. (TIFF) [file pbio.3000923.s004.tiff]

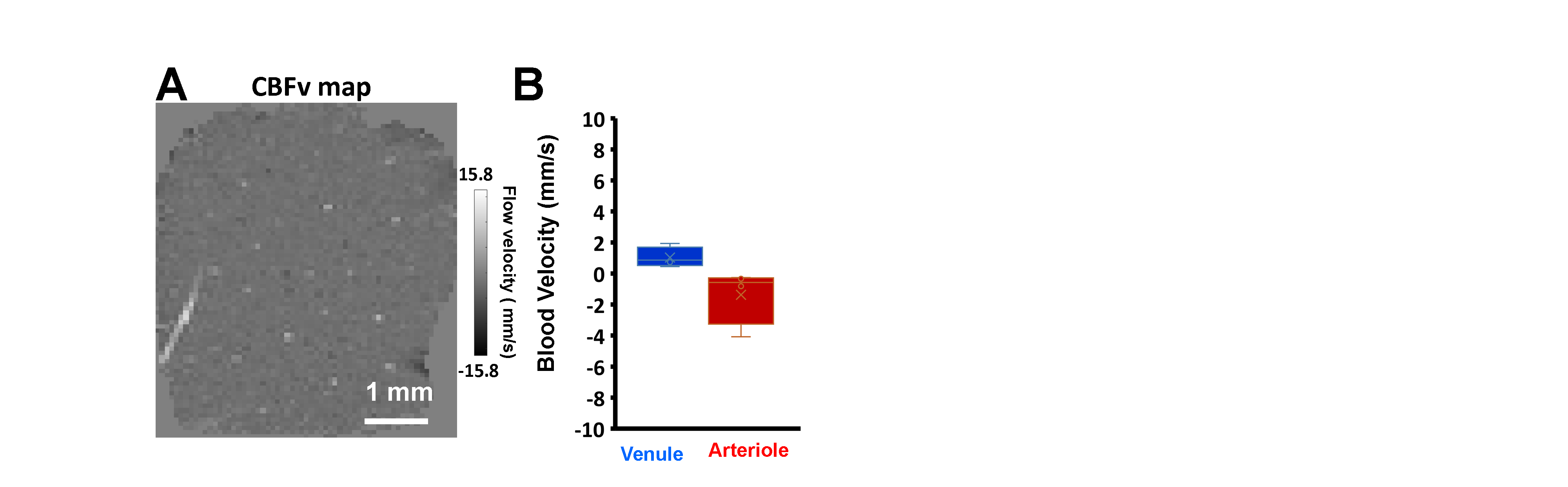

Supplement: S5 Fig — (A) The vectorized flow velocity (CBFv) map with 67 × 67 um2 in-plane resolution shows venules (bright dots) with positive CBFv values and arterioles (dark dots) with negative CBFv values. (B) The bar graph shows the averaged velocity for arterioles (−1.37 ± 0.94 mm/s) and venules (1.02 ± 0.32 mm/s) from 4 rats. The data underlying this figure can be found in S6 Data. CBFv, cerebral blood flow–related velocity; PC, phase contrast. (TIFF) [file pbio.3000923.s005.tiff]
