# Supplementary material for: Assessment of single-vessel cerebral blood velocity by phase contrast fMRI
Source: PLoS Biol. 2021 Sep 9;19(9):e3000923. doi: 10.1371/journal.pbio.3000923 (PMC8454982; doi:10.1371/journal.pbio.3000923)
Supplement: Attachment — Submitted filename: Response to reviewer_comments.docx [file pbio.3000923.s013.docx]

**Response to Reviewers**

We thank the valuable and insightful comments from all reviewers. Here, we have made new experiments and performed new data analysis based on reviewers’ comments. The following are the detailed point-to-point responses.

**Reviewer #1**: This paper is a follow up from their previous work which elegantly demonstrated single vessel BOLD and CBV -fMRI.   Here they extend this platform to include single vessel velocity measurements using high-resolution phase-contrast MRI.  This is a powerful technique with potential applications towards high-resolution imaging in humans and understanding hemodynamic responses in animal models.  The authors capture single vessel signals for BOLD, CBV and velocity across single trials.  I am enthusiastic about the study, but I do have a couple concerns.

1.	Flow and velocity are intermixed throughout the manuscript which is confusing.  I would prefer the authors avoid the use of flow to describe velocity. CBF is the quantity/time (eg. ml/min, RBCs/second) whereas velocity is the distance/time. I also suggest changing the name from CBF-fMRI to something else.

In the revised manuscript, we have changed CBF-fMRI to Cerebral Blood Flow-related velocity (CBFv) fMRI.

2.	Upon stimulation, it is surprising that the velocity seems to increase equally in both the arteriole and the veins.  Due to the arteriole specific volume increase, I would expect a smaller velocity change in the arteriole than the veins.  Previous work in olfactory bulb (PMID: 29937277), demonstrated such a phenomenon with two-photon measurements of vessel diameter and velocity.  Do the authors ever see mismatches between volume and velocity with their technique?

We agree with the reviewer. As shown in the revised Fig 3, quantitative analysis of the peak CBFv responses showed slightly higher velocity percentage changes in venules than arterioles (3.60 ± 0.80 % vs. 3.12 ± 0.87 %). We have highlighted this observation in results (Line 180-182). We also see the expected delay between the peak of arteriole versus venule flow - one sampling time (4 s) in the data of Figure E.

3.	Can the authors be confident the technique accurately reports absolute velocity?  The velocities they report seem very slow when compared to previous measurements of arteriole RBC velocity with two-photon scanning, (e.g. PMID: 19174826, Fig 1).  From Fig 2D it appears single arteriole velocities of >3mm/s were only observed in 1 of 6 rats and no arterioles had velocities above 5mm/s.  In Figure S2 Rat#2 has a mean arteriole velocity of <0.5mm/s (38 arterioles). Could there be contamination from tissue parenchyma, interstitial fluid, and surrounding capillary bed?  The authors state possible partial volume effects in the last sentence of the discussion.  But it would be important to clearly state how this may affect the quantitative measurements being made to avoid misinterpretation of the results.

We thank the reviewer to raise the variability issue of velocity measurement with the single-vessel PC method. Due to the spatial resolution (50 x 50 µm^2^) mismatch to the actual vessel size, the partial volume contribution will lead to estimation errors. We have discussed this issue (Line 146-152). Besides the partial volume, the variability of gradient performance and anesthetic effects across animals could also contribute to the error. In particular, we have realized that gradient accuracy is a crucial factor to better estimate the velocity. By optimizing the gradient performance during PC-MR scanning (see Method section, Line 371-384), we have acquired flow maps from another 5 rats. The revised Fig 2 shows mean velocity -3.7 ± 0.88 mm/s for arterioles and 2.59 ± 0.40 mm/s for venules from a total of 11 rats.

Minor:

Fig 2C, could the authors add a velocity scale bar.

L97 - typo, "while" matter

We have added the scale bar and corrected the typo.

**Reviewer #2**: This manuscript describes the use of phase contrast MRI (PC-MRI) for detecting velocity and velocity changes in signal cortical arteries and veins in the rat brain. High resolution PC MRI was performed at 14.1 T in anesthetized rats and compared to BOLD fMRI and contrast-enhanced CBV based fMRI for detecting functional activation during forepaw stimulation. Whereas BOLD fMRI was more sensitive to venules than arterioles and CBV MRI was more sensitive to arterioles than venules, PC-MRI sensitivity was comparable between venules and arterioles.  The authors conclude that the method will be useful for studying disease models and can be translated to human use for studying vascular dementia and other brain disorders.  While the methods and resulting data are reasonable, the main novelty of the work is carrying out PC-MRI at higher resolution than has been used previously while the rationale and implications of the method are poorly developed and its limitations are inadequately discussed. The manuscript could be improved by consideration of the following issues, all of which are essential to improving the work:

1.	The first sentence of the introduction describes CBF as a "key readout of neuronal processing." That is not strictly correct. Through neurovascular coupling, CBF is coupled to neural and not neuronal activity. It is less clear whether neuronal processing (spike patterns, etc.) are tightly linked to CBF changes.

We have revised the phrase to “a key hemodynamic readout coupled to neuronal dynamics” (Line 70).

2.	CBF generally refers to a quantifiable metric of flow, either in ml/min or ml/g/min. In the current work and much of the first paragraph of the introduction, the authors are referring to flow or changes in flow and not quantified CBF. For example, NIRS certainly does not measure CBF. It measures changes in oxyhemoglobin/deoxyhemoglobin ratios that may reflect underlying changes in CBF.

We thank the reviewer to raise the issue and we have corrected the sentence (Line 75).

3.	The discussion of flow sensitive methods leaves out vascular space occupancy (VASO) contrast.

We now discuss the vascular-space-occupancy (VASO) method as an endogenous measurements of cerebral blood volume (CBV) fMRI (Line 95).

4.	(line 95) The authors are incorrect that ASL cannot show orientation-specific effects.  Because continuous arterial spin labeling methods leverage directional flow, they contain directional information (see e.g. <https://pubmed.ncbi.nlm.nih.gov/26968145/>)

We thank the reviewer for raising the issue that the ASL-based flow measurement informs the directionality. We have revised the sentence and cite the corresponding reference (Line 97).

5.	(lines 141-1) All PC MRI is single vessel, so this sentence should be modified to contain information about the type of vessels.

We have revised the sentence accordingly (Line 154).

6.	(line 153) The manuscript describes results on "stimulation" without describing the nature of the stimulus. Although that is covered in the Methods, given the organization of the manuscript with the methods at the end, the Results should be more descriptive.

Detailed stimulus information is now included in the figure legend of Fig 3 (Line 688-689).

7.	Figure 3D is never described in the text of the manuscript. Figure 3E uses a different timing on the x-axis from 3C, which makes comparisons more difficult than it needs to be. The authors might also consider flipping the CBV and arteriolar plots in Figures B-E along the y-axis so that the changes show the same polarity as the other plots.

We now describe Fig 3D and adjusted the x-axis of Fig 3C as suggested by the reviewer. We kept the negative changes for the CBV-weighted signal from arterioles, as well as the velocity of arteriole blood, which were consistent with the functional maps shown in Fig 3A and supplementary movies.

8.	The supplemental movie is also difficult to follow.

We have revised the supplementary movie to better specify the venule-specific positive BOLD signal and arteriole-specific CBV-weighted signal, as well as the increase of positive CBFv in venules and decrease of negative CBFv in arterioles.

9.	Although a number of rats were studied with multiple contrast mechanisms, there does not appear to be any statistical treatment of the data.

We provide statistical analysis to show the histogram distribution of arterioles and venule CBFv values in Fig 2 and to compare peak BOLD, CBV, and CBFv responses between arterioles and venules. Detailed information is discussed in Figure legend of Fig 3

10.	The authors state in the Discussion and elsewhere that they have "optimized" PC-MRI in this work, but no optimization is presented.

We have added a section in method to better describe the detailed optimization steps for the single-vessel CBFv fMRI mapping (Line 371-384).

11.	One of the main weaknesses of this manuscript is the failure to address limitations of the PC-MRI approach. Firstly, because velocity encoding is directional in PC-MRI, it is sensitive to the angle of the vessel with respect to the velocity encoding gradient. Accordingly, the measured velocity changes will vary with this angle. While there may be literature demonstrating that cortical microvessels are largely orthogonal to the cortical surface, which could minimize such errors, this issue should be addressed. Even if it is true that cortical microvessels are orthogonal to the surface, the cortical surface is curved and the imaging slab is not. A second issue is that while imaging resolution is very high (50 microns) in-plane, the imaging slab is an order of magnitude larger. So, there are also likely significant partial volume effects in this approach. Both of these limitations undermine accurate quantification of flow changes.

We agree with the reviewer about the need for clarity on the limitations of CBFv. In the Discussion, we have highlighted the issues of partial volume effects on the blood velocity estimation using PC-MRI. We write:

“Another factor that contributes to the phase-dependent velocity error originates from the limited spatial resolution of PC-MRI images in comparison to the diameters of small vessels^1^, although corrections are possible^2, 3^. Given the parallel alignment of penetrating micro-vessels across cortical depth, we were able to identify individual micro-vessels in a 500 µm thickness slice perpendicular to the cortical surface. The different alignment angles of penetrating micro-vessels caused by the curvarture of cortex could also contribute to the velocity error.”

12.	Perhaps the biggest weakness of the manuscript is the failure to address the significance of PC-MRI versus existing methods for studying cortical function. While the authors demonstrate that PC-MRI is roughly equally sensitive to both arteriolar and venular flow changes, they don't say why this is advantageous for either basic or clinical research studies of regional brain function. They also do not address the challenges in translating this approach to human studies where fields strengths are lower, the cortical surface is further from the RF coil, and subjects are not paralyzed to minimize motion. Accordingly, there is zero basis in the manuscript for the conclusion that the method has translational potential for identifying vascular dementia, or what its potential advantages are as compared to existing methods. Indeed, based on Figure 3 it would appear that using PC-MRI to identify voxels of interest for BOLD contrast changes might provide superior data quality to dynamic PC-MRI. Thus, the potential to experimentally address a previously inaccessible biological question is not demonstrated in this work.

We have added a new paragraph to the Discussion to address the advantage of the PC-based CBFv-fMRI mapping in contrast to existing methods to study brain function. We write:

**“**The noninvasive measurement of micro-vessel blood velocity changes coupled to neuronal activation is a critical step to elucidate the neurovascular coupling mechanism underlying a variety of brain disorders in animal models. Existing methods, e.g. multi-photon microscopy^4-6^. or doppler-based ultrasound imaging**^7-9^**, enable the detection of red blood cell velocity or CBF/CBV from micro-vessels with ultra-high resolution, but craniotomy or thinned skull^10^ is typically needed for effective spectrum-specific signal transmission through deeper layers or subcortical regions in rats and other large animals. Such a surgical procedure has been well documented to induce perturbation of the physiological microenvironment in the brain^11-13^, which potentially confounds the disease-related functional CBF measurements in animal brains. In contrast, conventional MRI methods, including PC-MRI, mainly detect blood flow or velocity of large vessels, seldom identifying the functional CBFv of penetrating micro-vessels in the brain given the limited spatial and temporal resolution to maintain sufficient tSNR. By implementing a focal RF coil with the high field MR scanner, our work showed the feasibility to map the velocity changes from individual penetrating arterioles and venules with 20 to 70 µm^14, 15^.

The PC-based CBFv-fMRI provides a possible scheme to be combined with single-vessel BOLD and CBV fMRI signals for quantitative assessment of the cerebral metabolic rate of oxygen^16^. The vessel-specific hemodynamic measurements can be used to validate calibrated BOLD model^17^, probing the coupling of blood flow and energy metabolism in both normal and diseased animal brains in future studies. Furthermore, we tested the resting-state single-vessel correlation patterns of CBFv signals from penetrating arterioles and venules (Supplementary Fig 4). In contrast to the T2* weighted resting-state readout of BOLD or CBV signal fluctuation^15^, which amplifies the contrast-to-noise ratio (CNR) from the vessel-specific oxygenation or vessel diameter changes, CBFv signal fluctuation from penetrating microvessels has seldom been directly measured noninvasively across a large field-of-view. Here, we demonstrated a novel PC-based mapping scheme to measure the CBFv signal fluctuation from micro-vessels, which can be further combined with single-vessel CBV-fMRI to study the vasomotion-mediated perivascular clearance of animal models with Alzheimer’s disease^18, 19^. ”

Also, to highlight the novelty of our work, we have performed a new experiment to show the resting state single-vessel CBFv correlation patterns across penetrating arteriole and venules. This is new Supplemental Figure 4, shown below.

Supplementary Fig 4. The PC-based single-vessel resting-state CBFv-fMRI mapping.

A. The arteriole-venule (A-V) map shows bright dot as arterioles and dark dots as venules from one representative rat. Seed-based correlation maps were overlapped on the A-V map, showing two venule seeds (V1 and V2) and two arteriole seeds (A1 and A2). The venule-based correlation map shows positive correlations to other venules, but negative correlations with arterioles. In contrast, the arteriole-based correlation map shows positive correlations to other arterioles, but negative correlations with venules.

B. The enlarged CBFv map shows the venule with positive velocity (bright dots) and arterioles with negative velocity (dark dots). The venule seed-based CBFv correlation map was overlapped on the CBFv map, showing the positive correlation on surrounding venule voxels (bright dots), and negative correlation on surrounding arteriole voxels (dark dots).

C. The normalized time course extracted from venule and arteriole voxels, showing correlated low-frequency signal fluctuation.

D. The power spectrum density (PSD) plot of resting state CBFv dynamics from arteriole and venule voxels shows the slightly higher power from 0.01 to 0.04Hz (n=5).

**Reviewer #3**: In this work the authors have implemented and evaluated a magnetic resoance imaging (MRI) method to measure cerebral blood flow (CBF) with singel vessel resolution in the rat brain, based on the phase contrast (PC) technique. This method complements and is a valuable addition to the established BOLD and CBV techniques to assess brain function by MRI. The authors have previously pioneered single vessel fMRI, and now combine their recent methods with PC-MRI. The novel method is a valuable asset for functional brain imaging and may help to further understand mechanisms of neurovascular coupling and brain function in general.

The work is carefully conducted, the manuscript well written, and of great relevance and interest to the fMRI neuroimaging community. However, for a wider readership, the paper may be too detailed.

While I have no major points that would have to be addressed before the paper can be published, I think a few clarifications concerning reproducibility and possible applications are necessary:

1. The measurements were conducted at 14.1 tesla, a magnetic field strength that is not available in most labs. Will this method also be feasible at the more common 7 tesla or 9.4 tesla?

We have tested the PC-based single vessel velocity mapping using 9.4 T MR scanner. Supplementary Fig. 5 shows the feasibility to detect the CBFv from individual arterioles and venules with velocity -1.37 ± 0.91 mm/s for arterioles and 1.02 ± 0.32 mm/s for venules. We have also discussed the broader usage of PC-based single vessel velocity mapping (Line 259-262).

2. Which pulse program/MR sequence did the authors use; was it the sequence provided by the vendor Bruker? 3. For replication of these experiments, details such as the exact sequence and gradient timing (and ramps) are important. Is it possible to share these details perhaps even the source code of the method (in a supplement)?

We answer questions 2 and 3 together. Yes. The basic pulse sequence is provided by the vendor Bruker. We have optimized the sequence for the single-vessel CBFv fMRI. In the Method section, we provide detailed parameter optimization of the PC-based flow mapping method (Line 371-384) and the source code can be directly procured through the Bruker console.

4. Are the data presented in Fig. 2D values from single voxels or were they averaged (over how many voxels)? Figure S3 creates the impression that single vessels spread over five to nine voxels.

The vessel voxel was identified based on the arteriole-venule (A-V) map. We have described the definition of the vessel voxels in the Method section. Based on the 50 x 50um^2^ in-plane resolution, vessel voxels usually covered 2-6 voxels. Fig S3 overlaid the single-vessel fMRI functional maps on the A-V map, which was resampled to 50 x 50um^2^ in-plane resolution. The BOLD/CBV maps rely on the T2* weighted signals, extending to 5-9 voxels, but CBFv maps are mostly restrained to be less than 5-6 voxels.

5. What is the minimum vessel diameter that can be analyzed with the presented method? How would this be different at other field strengths or in humans? What was the average vessel diameter in this study?

In our previous work (Yu et al. Nature Methods, 2016; He et al. Neuron, 2018), we have identified the vessel diameter for single-vessel mapping ranges from 20 to 70 µm. We have applied the same single-vessel mapping scheme to acquire the A-V map in this work. As shown in Fig 2, the A-V map is well overlapped with the CBFv map from individual vessels with a minimum diameter of around 20 µm.

While not fundamentally impossible, it is currently not feasible for human brain mapping to image vessels with this resolution given the limited gradient strength and magnetic field strength. Yet, as shown previously (He et al Neuron, 2018), the A-V map of the human cortex detects the sulcus vein and artery. Finer resolution represents work in program by our MGH colleague Jonathan Polimeni.

6. In line 210 the author speculate on a translational potential. How can his method be translated? Is single vessel-CBF feasible in humans? What field strength would be required?

We thank the reviewer to raise this issue. We have revised this sentence: “Previously, we have demonstrated the feasibility of the single-vessel BOLD fMRI to target sulcus vein and artery in the occipital lobe of the human brain^15^, providing a potential direction of PC-based CBFv human brain mapping.

In contrast to the BOLD effects, the pulsation could be a main confounding issue for the CBFv functional mapping^20, 21^. The translational potential of the PC-based single vessel CBFv mapping needs to be further evaluated with a better design of the phased-array surface coil for focal field-of-view measurement and accelerated PC-MRI sequence.”

Typos:

l97: "white matter"

We have corrected it.

l275: missing "were"

Legend to Fig. 3: check for use of direct articles.

We have corrected it.

**References**

1.	Geurts LJ, Bhogal AA, Siero JCW, Luijten PR, Biessels GJ, Zwanenburg JJM. Vascular reactivity in small cerebral perforating arteries with 7T phase contrast MRI - A proof of concept study. NeuroImage. 2018;172:470-7. Epub 2018/02/07. doi: 10.1016/j.neuroimage.2018.01.055. PubMed PMID: 29408324; PMCID: PMC5915583.

2.	Hoogeveen RM, Bakker CJ, Viergever MA. MR phase-contrast flow measurement with limited spatial resolution in small vessels: value of model-based image analysis. Magnetic resonance in medicine : official journal of the Society of Magnetic Resonance in Medicine / Society of Magnetic Resonance in Medicine. 1999;41(3):520-8. Epub 1999/04/16. doi: 10.1002/(sici)1522-2594(199903)41:3<520::aid-mrm14>3.0.co;2-a. PubMed PMID: 10204875.

3.	Zong X, Lin W. Quantitative phase contrast MRI of penetrating arteries in centrum semiovale at 7T. NeuroImage. 2019;195:463-74. Epub 2019/04/03. doi: 10.1016/j.neuroimage.2019.03.059. PubMed PMID: 30935910.

4.	Kleinfeld D, Mitra PP, Helmchen F, Denk W. Fluctuations and stimulus-induced changes in blood flow observed in individual capillaries in layers 2 through 4 of rat neocortex. Proceedings of the National Academy of Sciences of the United States of America. 1998;95(26):15741-6. PubMed PMID: 9861040; PMCID: PMC28114.

5.	Demirhan Kobat1, Michael E. Durst1, Nozomi Nishimura2, Angela W. Wong2, Chris B. Schaffer2, and Chris Xu1. Deep tissue multiphoton microscopy using longer wavelength excitation2009.

6.	Kobat D, Horton NG, Xu C. In vivo two-photon microscopy to 1.6-mm depth in mouse cortex. J Biomed Opt. 2011;16(10):106014. Epub 2011/10/28. doi: 10.1117/1.3646209. PubMed PMID: 22029361.

7.	Mace E, Montaldo G, Cohen I, Baulac M, Fink M, Tanter M. Functional ultrasound imaging of the brain. Nature methods. 2011;8(8):662-4. doi: 10.1038/nmeth.1641. PubMed PMID: 21725300.

8.	Sieu LA, Bergel A, Tiran E, Deffieux T, Pernot M, Gennisson JL, Tanter M, Cohen I. EEG and functional ultrasound imaging in mobile rats. Nature methods. 2015;12(9):831-4. Epub 2015/08/04. doi: 10.1038/nmeth.3506. PubMed PMID: 26237228; PMCID: PMC4671306.

9.	Rungta RL, Osmanski BF, Boido D, Tanter M, Charpak S. Light controls cerebral blood flow in naive animals. Nature communications. 2017;8:14191. Epub 2017/02/01. doi: 10.1038/ncomms14191. PubMed PMID: 28139643; PMCID: PMC5290324.

10.	Drew PJ, Shih AY, Driscoll JD, Knutsen PM, Blinder P, Davalos D, Akassoglou K, Tsai PS, Kleinfeld D. Chronic optical access through a polished and reinforced thinned skull. Nat Methods. 2010;7(12):981-4. Epub 2010/10/23. doi: 10.1038/nmeth.1530. PubMed PMID: 20966916; PMCID: PMC3204312.

11.	Xu HT, Pan F, Yang G, Gan WB. Choice of cranial window type for in vivo imaging affects dendritic spine turnover in the cortex. Nature neuroscience. 2007;10(5):549-51. Epub 2007/04/10. doi: 10.1038/nn1883. PubMed PMID: 17417634.

12.	Dorand RD, Barkauskas DS, Evans TA, Petrosiute A, Huang AY. Comparison of intravital thinned skull and cranial window approaches to study CNS immunobiology in the mouse cortex. Intravital. 2014;3(2). Epub 2015/01/09. doi: 10.4161/intv.29728. PubMed PMID: 25568834; PMCID: PMC4283137.

13.	Cole JT, Yarnell A, Kean WS, Gold E, Lewis B, Ren M, McMullen DC, Jacobowitz DM, Pollard HB, O'Neill JT, Grunberg NE, Dalgard CL, Frank JA, Watson WD. Craniotomy: true sham for traumatic brain injury, or a sham of a sham? Journal of neurotrauma. 2011;28(3):359-69. Epub 2010/12/31. doi: 10.1089/neu.2010.1427. PubMed PMID: 21190398; PMCID: PMC3057208.

14.	Yu X, He Y, Wang M, Merkle H, Dodd SJ, Silva AC, Koretsky AP. Sensory and optogenetically driven single-vessel fMRI. Nature methods. 2016;13(4):337-40. doi: 10.1038/nmeth.3765. PubMed PMID: 26855362.

15.	He Y, Wang M, Chen X, Pohmann R, Polimeni JR, Scheffler K, Rosen BR, Kleinfeld D, Yu X. Ultra-Slow Single-Vessel BOLD and CBV-Based fMRI Spatiotemporal Dynamics and Their Correlation with Neuronal Intracellular Calcium Signals. Neuron. 2018;97(4):925-39 e5. Epub 2018/02/06. doi: 10.1016/j.neuron.2018.01.025. PubMed PMID: 29398359; PMCID: PMC5845844.

16.	Davis TL, Kwong KK, Weisskoff RM, Rosen BR. Calibrated functional MRI: mapping the dynamics of oxidative metabolism. Proceedings of the National Academy of Sciences of the United States of America. 1998;95(4):1834-9. Epub 1998/03/21. doi: 10.1073/pnas.95.4.1834. PubMed PMID: 9465103; PMCID: PMC19199.

17.	Leontiev O, Dubowitz DJ, Buxton RB. CBF/CMRO2 coupling measured with calibrated BOLD fMRI: sources of bias. NeuroImage. 2007;36(4):1110-22. Epub 2007/05/26. doi: 10.1016/j.neuroimage.2006.12.034. PubMed PMID: 17524665; PMCID: PMC2038985.

18.	Mateo C, Knutsen PM, Tsai PS, Shih AY, Kleinfeld D. Entrainment of Arteriole Vasomotor Fluctuations by Neural Activity Is a Basis of Blood-Oxygenation-Level-Dependent "Resting-State" Connectivity. Neuron. 2017;96(4):936-48 e3. doi: 10.1016/j.neuron.2017.10.012. PubMed PMID: 29107517; PMCID: PMC5851777.

19.	van Veluw SJ, Hou SS, Calvo-Rodriguez M, Arbel-Ornath M, Snyder AC, Frosch MP, Greenberg SM, Bacskai BJ. Vasomotion as a Driving Force for Paravascular Clearance in the Awake Mouse Brain. Neuron. 2020;105(3):549-61 e5. Epub 2019/12/08. doi: 10.1016/j.neuron.2019.10.033. PubMed PMID: 31810839; PMCID: PMC7028316.

20.	Wagshul ME, Eide PK, Madsen JR. The pulsating brain: A review of experimental and clinical studies of intracranial pulsatility. Fluids Barriers CNS. 2011;8(1):5. Epub 2011/02/26. doi: 10.1186/2045-8118-8-5. PubMed PMID: 21349153; PMCID: PMC3042979.

21.	Driver ID, Traat M, Fasano F, Wise RG. Most Small Cerebral Cortical Veins Demonstrate Significant Flow Pulsatility: A Human Phase Contrast MRI Study at 7T. Frontiers in neuroscience. 2020;14:415. Epub 2020/05/21. doi: 10.3389/fnins.2020.00415. PubMed PMID: 32431591; PMCID: PMC7214844.
