# Supplementary material for: Assessment of single-vessel cerebral blood velocity by phase contrast fMRI
Source: PLoS Biol. 2021 Sep 9;19(9):e3000923. doi: 10.1371/journal.pbio.3000923 (PMC8454982; doi:10.1371/journal.pbio.3000923)
Supplement: Attachment — Submitted filename: Responses to reviewer comments_0720.docx [file pbio.3000923.s014.docx]

Responses to reviewer comments:

We thank the valuable comments from Reviewer #2. We have made the point-to-point responses based on reviewer comments.

*Reviewer #2.*

*The revised manuscript has addressed many of my concerns, and is also supported by new data. The findings of this study remain interesting. However, some of the modifications to the manuscript still need to be revised for clarity. The manuscript also needs careful editing for English syntax.

1.      Line 221: "focal" RF coil should be "local" RF coil*

We have corrected it.

*2.      Line 223:  "diameter" (or radius) is missing from this sentence*

We have corrected it.

*3.      Line 230-234: The paragraph beginning "In contrast to the T2* weighted resting-state" leads the reader to expect something about differing contrast mechanisms, but the sentence winds up being about frequency (CBFv signal fluctuation … has seldom been directly measured …"  Why is this statement in the middle of a paragraph about MRI-based CMRO2 measurements? Is there really a need to mention that their novel approach has been used less often than the most widely used approach?*

We thank the comments. Now, we have rewritten this paragraph to highlight “The novel applications of PC-based CBFv-fMRI can be outlined in two different directions.” Also, we have revised the sentence of “CBFv signal fluctuation from penetrating micro-vessels provides a direct measurement of blood velocity changes noninvasively across a large field-of-view”.

*4.      Lines 234-237: Then this same paragraph concludes with a sentence suggesting that this approach can be used to study glymphatic function without explaining how. Paragraphs should have clear themes.*

We have revised this sentence with “The measurement of PC-based CBFv signal fluctuation from micro-vessels can be further combined with single-vessel CBV-fMRI to study the vascular dynamic mechanism that underlies vasomotion-mediated perivascular clearance in animal models with Alzheimer’s disease [59, 60]. In particular, both vasomotion-based ateriole diameter changes, i.e., the CBV signal fluctuation, and corresponding blood velocity changes, i.e. CBFv signal fluctuation, can be mapped across hippocampal penetrating vessels. This may be further combined with simultaneous fiber photometry-base fluorescent recordings from genetically encoded biosensors [39] to identify the pathological vasodynamics in transgenic AD animals.”

*5.      Line 238: The next paragraph switches to hardware limitations of phantom studies, but without clearly indicating that except through saying "phantom capillary tubing". Paragraphs should have clear themes.*

We have revised this paragraph with “The accurate measurement of PC-based CBFv from micro-vessels relies on multiple factors. First, a remaining complication with PC-MRI mapping is the presence of small offsets in velocity as shown in our phantom capillary tubing studies with circulating flow under different conditions.”

*6.      Lines 251-253: This sentence seems to be addressing partial volume effects, but doesn't ever say that directly.*

We have specified the “partial volume effect” in this sentence as “Another factor that contributes to the phase-dependent velocity error originates from the limited spatial resolution of PC-MRI images in comparison to the diameters of small vessels, i.e. the partial volume effect [33], although corrections are possible[27, 34].”

*7.      Lines 258-267: This paragraph appears to be an effort to respond to the need for better description of the translational (to humans) potential of this method, but the first two sentences discuss animal studies at 9.4T, the third sentence discusses a different type of human functional PC study. The fourth sentence mentions "the pulsation" as a potential confound without explaining what that is, and the last sentence mentions some technical challenges without explaining why they are important.*

We thank the reviewer to highlight the issue. We have revised this paragraph with “The present work shows the feasibility of PC-based CBFv-fMRI in rat brains using a ultra-high field strength MR scanner (14 T). It should be noted that the high-resolution PC-based CBFv measurement can also be applied with 9.4 T scanner to detect the micro-vessel blood velocity at a similar scale to 14 T measurement (**Supplementary Figure 5**). This supports a broader usage of noninvasive animal CBFv mapping. The translational potential of the PC-based CBFv fMRI remains to be investigated. Previously, we detected low-frequency fluctuation of single-vessel resting state BOLD fMRI signals (TR = 1 s) from individual sulcus veins and arteries in the occipital lobe of the human brain [38], bearing the interference of pulsation and other motion effects. Different from the T2*-weighted BOLD effects, pulsation could be a significant confounding issue for PC-based blood velocity mapping from individual vessels [61, 62]. To better differentiate the pulsational contribution to CBFv signal fluctuation given different frequency ranges, we will need to increase the sampling rate by implementing phased-array surface coils for focal field-of-view measurement and accelerated PC-MRI sequence.”

*8.      Also, in response to Reviewer 1, the authors have replaced "CBF-fMRI" to "CBFv" which is at best minimally better that CBF-fMRI in differentiating the PC MRI measure (velocity) from CBF.*

We agree with the reviewer.
